# Effect of Drying and Cooking on the Chemical Composition, Phenolic Profile, and Antioxidant Capacity of *Chenopodium berlandieri* subsp. *nuttalliae*: A Metabolomic Approach

**DOI:** 10.3390/plants15091366

**Published:** 2026-04-29

**Authors:** Ángel Félix Vargas-Madriz, Perla del Carmen Bautista-Cano, Carlos Vázquez Jiménez, Jenny Kay Novella-Erreguín, Haidel Vargas-Madriz, Aarón Kuri-García, Iza Fernanda Pérez-Ramírez, Roberto Augusto Ferriz-Martínez, Karina de la Torre-Carbot, Carlos Saldaña, Jorge Luis Chávez-Servín

**Affiliations:** 1 Department of Cell and Molecular Biology, Facultad de Ciencias Naturales, Universidad Autónoma de Querétaro, Santiago de Querétaro 76230, Mexico; angel.vargas@uaq.mx (Á.F.V.-M.); perla.bautista.cano@gmail.com (P.d.C.B.-C.); cvazquez26@alumnos.uaq.mx (C.V.J.); jnovella09@alumnos.uaq.mx (J.K.N.-E.); aaron.kuri@uaq.mx (A.K.-G.); roberto.augusto.ferriz@uaq.mx (R.A.F.-M.); karina.delatorre@uaq.edu.mx (K.d.l.T.-C.); 2 Department of Agricultural Production, University Center of the South Coast, Universidad de Guadalajara, Av. Independencia Nacional, No 151, Ciudad de Guadalajara 48900, Mexico; haidel_vargas@hotmail.com; 3 Facultad de Química, Universidad Autónoma de Querétaro, Cerro de las Campanas S/N, Santiago de Querétaro 76010, Mexico; iza.perez@uaq.mx; 4 Membrane Biophysics and Nanotechnology Laboratory, Facultad de Ciencias Naturales, Universidad Autónoma de Querétaro, Av. De las Ciencias s/n, Juriquilla, Santiago de Querétaro 76230, Mexico; carlos.saldana@uaq.mx

**Keywords:** *Chenopodium berlandieri* subsp. *nuttalliae*, huauzontle, phenolic compounds, antioxidant capacity, freeze-drying and oven-drying, thermal processing, metabolomics

## Abstract

Traditional edible plants such as quelites are an important component of the Mexican diet due to their nutritional and functional value; however, the effects of postharvest and culinary processing on their phytochemical composition remain poorly understood. This study evaluated the impact of oven-drying and freeze-drying, as well as thermal preparation (raw vs. boiled), on the proximal chemical composition, phenolic profile, and antioxidant capacity of leaves and inflorescences of *Chenopodium berlandieri* subsp. *nuttalliae* (huauzontle), using an integrated metabolomic approach. Proximal analysis showed that major macronutrients (protein, dietary fiber, lipids, and carbohydrates) were largely preserved across drying methods, whereas moisture and ash contents differed significantly among tissues and treatments (*p* < 0.05). Raw freeze-dried inflorescences exhibited the highest total phenolic content and antioxidant capacity. UPLC-DAD-ESI-QToF/MS enabled the identification and quantification of 26 phenolic compounds, predominantly glycosylated flavonols derived from quercetin, kaempferol, and isorhamnetin, while naringin was identified as the main flavanone glycoside present. Quercetin glucuronide was the most abundant compound, particularly in inflorescences. Multivariate analyses (principal component analysis [PCA], permutational multivariate analysis of variance [PERMANOVA], and partial least squares discriminant analysis [PLS-DA]) suggested that the drying method was a major source of variability, followed by thermal treatment and tissue type, although these patterns should be interpreted as indicative rather than conclusive. Overall, freeze-drying appeared to be the most effective method for preserving the phytochemical quality of huauzontle under the conditions evaluated, highlighting its potential as a valuable source of bioactive compounds within the genus *Chenopodium*.

## 1. Introduction

Traditionally, Mexico hosts a wide diversity of plant species that have been used for both food and medicinal purposes, among which *quelites* stand out. The term *quelite* derives from the Nahuatl word *quilitl*, meaning “edible tender plant” [1,2]. In Mexico, the consumption of approximately 500 plant species classified as *quelites* has been documented. These native plants are mainly characterized as young annual herbs, as well as inflorescences, flowers, and shoot tips of perennial species. Traditionally, *quelites* are consumed fresh or subjected to various culinary preparations, including boiling, stewing, roasting, frying, or steaming, and they are frequently used as ingredients in a wide variety of regional dishes [3]. Representative species include *Amaranthus* spp. (quintonil), *Chenopodium* spp. (quelite cenizo), and *Portulaca oleracea* (verdolaga), which have long been incorporated into traditional Mexican gastronomy, contributing flavor, aroma, and color, while also providing important nutritional value due to their content of proteins, dietary fiber, vitamins, minerals, and bioactive compounds [4].

*Chenopodium berlandieri* subsp. *nuttalliae* (Saff.) H.D. Wilson & Heiser (commonly known as “huauzontle”) is a traditional edible plant belonging to the family Amaranthaceae, native to Mexico and widely consumed as a quelite [5]. This taxon is part of the *C. berlandieri* species complex, which is characterized by high morphological variability and taxonomic complexity [6]. Its taxonomic status is currently under revision. According to Plants of the World Online (POWO), *C. berlandieri* subsp. *nuttalliae* is treated as a synonym of *C. berlandieri* var. *berlandieri*, whereas World Flora Online (WFO) considers it a synonym of *C. berlandieri Moq*. Despite these discrepancies, the designation *C. berlandieri* subsp. *nuttalliae* is widely used in ethnobotanical, agronomic, and nutritional studies in Mexico, where it is recognized as a domesticated form within the *C. berlandieri* complex [5]. Previous studies have described this taxon as a semi-domesticated or domesticated lineage derived from wild populations of *C. berlandieri* through human selection in North America [7]. In this context, *C. berlandieri* subsp. *nuttalliae* is closely related to *C. berlandieri* subsp. *berlandieri*, which represents the wild or weedy counterpart within the same species complex. Although both taxa share a common evolutionary origin, they differ in their degree of domestication, morphological traits, ecological adaptation, and potentially in their phytochemical composition. Therefore, in the present study, the nomenclature *C. berlandieri* subsp. *nuttalliae* was retained to ensure consistency with previous literature and regional usage, while explicitly acknowledging its synonymy and current taxonomic considerations.

In several rural regions of the country, both the leaves and inflorescences are consumed in a variety of traditional dishes [8,9]. This species has gained increasing attention due to its nutritional and medicinal attributes. Previous studies have reported significant levels of macronutrients such as protein, dietary fiber, and carbohydrates, as well as essential minerals including sodium, phosphorus, magnesium, potassium, calcium, and iron [10]. Overall, *quelites* have been shown to exhibit higher nutritional value than conventional leafy vegetables such as lettuce and spinach, making them an accessible and affordable food source [11,12].

In addition, colorimetric methods have been used to report total phenolic compounds, total flavonoids, and condensed tannins in *C. berlandieri* [10,13], however, to date, individual phenolic compounds in different tissues of *C. berlandieri* have not been comprehensively characterized. In other *quelite* species such as papalo (*Porophyllum ruderale*), chaya (*Cnidoscolus aconitifolius*), and *C. berlandieri* subsp. *berlandieri* (quelite cenizo), various phenolic acids (e.g., ferulic, caffeic, and chlorogenic acids) and flavonoids (e.g., catechin, naringin, quercetin, kaempferol, and apigenin) have been identified, with marked interspecific variability [14,15]. Phenolic compounds are widely associated with the prevention and management of chronic non-communicable diseases due to their biological properties, including antioxidant, anti-inflammatory, antimicrobial, antifungal, antimutagenic, and antiproliferative activities [4]. Nevertheless, the phenolic profile of plant-based foods can vary substantially depending on postharvest processing and thermal treatments, such as drying and boiling, which may promote either degradation or release of free and conjugated phenolics from the plant matrix [16].

Drying is an essential process for preserving plant materials prior to analysis and has been used since ancient times for food conservation. This process involves the removal of water to prevent enzyme activation and microbial growth that can compromise overall food quality [17]. Several drying techniques have been reported in the scientific literature, including methods based on thermal treatments such as sun-drying, oven-drying, and microwave-drying, which operate under different temperatures and drying times. In contrast, freeze-drying represents another widely applied technique for dehydrating plant materials [16]. Sun-drying is the oldest drying method and relies on natural climatic conditions, requiring minimal energy input; however, it involves prolonged exposure times and is highly dependent on environmental factors. These limitations can reduce the efficiency of the process and promote the degradation of phytochemical compounds due to fluctuations in temperature and relative humidity, as well as potential microbial growth [17,18].

Among postharvest processing methods, oven-drying is one of the most frequently reported techniques for drying and storage of plant samples, as it allows for greater process control through the use of hot air. Nevertheless, this method often requires extended drying periods and elevated temperatures, which may lead to the degradation or reduction in bioactive compounds and induce alterations in the physicochemical characteristics of plant materials [19].

In contrast, freeze-drying is a dehydration process carried out under low temperature and reduced pressure, in which frozen water is removed by sublimation. This drying method offers the advantage of preserving the physicochemical characteristics of plant materials, mainly due to the freezing step, which contributes to the inactivation of enzymatic activity and microbial growth [17]. Freeze-drying has been reported to effectively preserve thermolabile antioxidant compounds, including phenolic compounds, ascorbic acid, carotenoids, and tocopherols; however, it is also considered one of the most expensive drying techniques, requiring specialized equipment and higher operational costs [20].

Another thermal process commonly applied in the preparation of *quelites* is cooking, with boiling being the most widely used hydrothermal treatment. Boiling is traditionally employed to improve flavor and enhance organoleptic properties; nevertheless, this culinary process may negatively affect the phenolic profile of foods [21,22]. Conversely, other studies have shown that boiling can enhance the extractability of phenolic compounds bound to the plant matrix, although this effect largely depends on the specific constituents of each plant species [15]. Therefore, the aim of this study was to evaluate the impact of two drying methods (oven-drying and freeze-drying) and thermal preparation (raw and boiled) on the proximal chemical composition, phenolic profile, and antioxidant capacity of leaves and inflorescences of *C. berlandieri* subsp. *nuttalliae*. Specifically, this work aimed to (i) assess changes in macronutrient composition, (ii) quantify antioxidant capacity using colorimetric assays (2,2-diphenyl-1-picrylhydrazyl [DPPH], ferric reducing antioxidant power [FRAP], and 2,2′-azino-bis(3-ethylbenzothiazoline-6-sulfonic acid) [ABTS]), and (iii) identify and quantify individual phenolic compounds using ultra-performance liquid chromatography coupled to diode array detection and electrospray ionization quadrupole time-of-flight mass spectrometry (UPLC-DAD-ESI-QToF/MS).

## 2. Results

### 2.1. Proximal Chemical Composition of C. berlandieri subsp. nuttalliae

The proximal chemical composition of leaves and inflorescences of *C. berlandieri* subsp. *nuttalliae* showed variations depending on tissue type and drying method (Table 1).

Significant differences (*p* < 0.05) were observed in moisture, ash, and lipid contents across tissues and drying treatments. Inflorescences exhibited a significantly higher lipid content (*p* < 0.05) under both drying methods, whereas leaves showed a significantly higher ash content (*p* < 0.05) compared to inflorescences. Protein content did not differ significantly (*p* > 0.05) between tissues when the same drying method was applied (freeze-dried inflorescences vs. freeze-dried leaves; oven-dried inflorescences vs. oven-dried leaves). Similarly, no significant differences (*p* > 0.05) were detected in dietary fiber or total carbohydrate contents between tissues or drying treatments. Moisture content remained below 15% in both tissues regardless of the drying method, indicating effective dehydration under the conditions evaluated.

Overall, these results indicate that drying method and tissue type mainly influenced moisture, ash, and lipid contents, whereas protein, fiber, and carbohydrate levels remained relatively stable across treatments.

### 2.2. Phenolic Content and Antioxidant Capacity of C. berlandieri subsp. nuttalliae

The results obtained from colorimetric assays for total phenolic content (TPC), total flavonoid content (TFC), condensed tannins (CT), and antioxidant capacity (DPPH, FRAP, and ABTS) in leaves and inflorescences of *C. berlandieri* subsp. *nuttalliae* subjected to oven-drying and freeze-drying, as well as thermal treatment (raw and boiled), are summarized in Table 2.

Regarding TPC, raw freeze-dried inflorescences (I_FD_R) exhibited the highest values (30.42 ± 1.72 mg GAE/g DE), which were significantly higher (*p* < 0.05) than those observed in oven-dried and boiled samples. In contrast, oven-dried boiled inflorescences (I_OD_B) showed the lowest TPC values (17.98 ± 1.89 mg GAE/g DE).

Leaf samples showed intermediate TPC values, ranging from 24.08 ± 1.75 to 27.03 ± 3.77 mg GAE/g DE. Raw oven-dried leaves (L_OD_R) showed the highest TPC values among leaf treatments, although no significant differences were observed compared with boiled freeze-dried leaves (L_FD_B).

No significant differences (*p* > 0.05) in TFC were observed between raw oven-dried (I_OD_R) and raw freeze-dried (I_FD_R) inflorescences. However, boiling significantly reduced TFC in oven-dried inflorescences (I_OD_B), whereas freeze-dried boiled samples (I_FD_B) showed TFC values comparable to those of I_FD_R. In leaves, TFC did not differ significantly between raw samples dried by either method, while boiled samples showed a significant reduction (*p* < 0.05), particularly in oven-dried leaves (L_OD_B).

Condensed tannin content differed significantly among treatments. The highest CT concentration was observed in raw oven-dried inflorescences (I_OD_R; 1.08 ± 0.06 mg CE/g DE). In leaves, raw samples exhibited higher CT values, whereas boiling resulted in a significant reduction. No significant differences were observed between raw and boiled freeze-dried inflorescences (I_FD_R and I_FD_B).

Antioxidant capacity assessed by the DPPH assay showed that raw inflorescences exhibited significantly higher radical scavenging activity under both drying methods (*p* < 0.05). In contrast, boiled freeze-dried leaves (L_FD_B) exhibited the lowest antioxidant capacity. Similar trends were observed using the ABTS assay. Raw oven-dried (I_OD_R; 564.35 ± 17.54 µM TE/g DE) and raw freeze-dried inflorescences (I_FD_R; 604.85 ± 18.65 µM TE/g DE) showed higher antioxidant capacity than their boiled counterparts. In leaves, raw oven-dried samples exhibited higher ABTS values, which decreased significantly after boiling (*p* < 0.05), whereas freeze-dried leaves showed higher ABTS values after boiling compared to raw samples. Results obtained using the FRAP assay showed a similar trend to that observed for the other antioxidant assays. In inflorescences, raw samples exhibited significantly higher reducing capacity than boiled samples under both drying methods (*p* < 0.05). In leaf tissues, FRAP values were moderately higher in freeze-dried samples than in oven-dried ones, suggesting a greater preservation of reducing capacity under freeze-drying conditions. Consistently, freeze-dried inflorescences showed significantly higher FRAP values than oven-dried samples.

**Figure 1:**
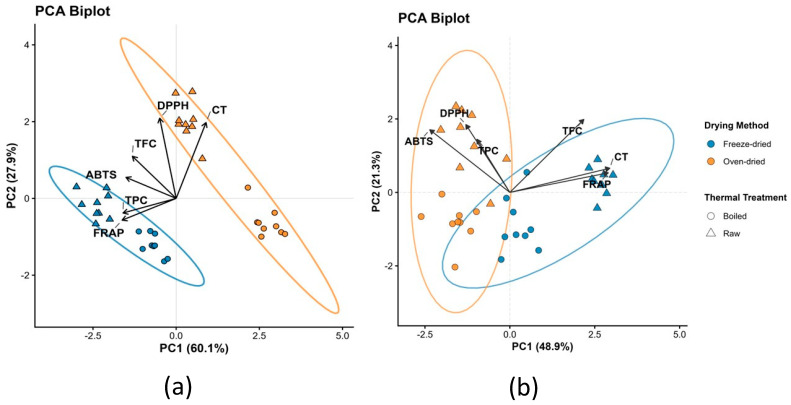
Principal component analysis (PCA) biplots illustrating the distribution of samples based on phenolic composition and antioxidant capacity in (**a**) inflorescences and (**b**) leaves subjected to different drying methods and thermal treatments. Colors represent drying methods (blue: freeze-dried; orange: oven-dried), while symbol shapes indicate thermal treatment (circles: boiled; triangles: raw). Each point represents the mean value of each experimental treatment (*n* = 9 analytical replicates per treatment).

Principal component analysis (PCA) suggested a separation trend of inflorescence samples according to drying method (Figure 1a). Thermal treatments are represented by symbol shape (circles for boiled and triangles for raw samples), facilitating interpretation of treatment effects. The first principal component (PC1) explained 60.1% of the total variance and indicated differentiation patterns between freeze-dried and oven-dried samples, which were located on the negative side of PC1, from oven-dried samples positioned on the positive side. Variables with the strongest negative loadings on PC1 included FRAP (−0.5077), TPC (−0.4990), and ABTS (−0.4709), indicating that higher values of these parameters were associated with freeze-dried samples. In contrast, DPPH (+0.6530) and condensed tannins (CT; +0.6174) showed positive loadings and were more closely associated with oven-dried samples. The second principal component (PC2), explaining 27.9% of the variance, reflected a secondary separation related to thermal treatment, with raw samples generally showing higher PC2 scores than boiled samples. The PCA approach allows simultaneous visualization of multiple experimental factors, preserving the interaction between drying method and thermal treatment within each tissue.

PCA of leaf samples suggested a similar separation pattern, primarily associated with drying method (Figure 1b). PC1 explained 48.9% of the variance, separating freeze-dried (negative loadings) from oven-dried samples (positive loadings). PC2 explained 21.3% of the variance and was mainly associated with thermal treatment.

PERMANOVA analysis confirmed statistically significant multivariate effects of tissue type, drying method, thermal treatment, and their interactions on the colorimetric and antioxidant variables evaluated (TPC, TFC, CT, DPPH, FRAP, and ABTS) (*p* < 0.001 for all terms; Table 3). The interaction between tissue type and drying method explained the largest proportion of variance (R^2^ = 0.25687), followed by drying method (R^2^ = 0.16311) and thermal treatment (R^2^ = 0.17608), indicating that both plant tissue and postharvest processing contributed substantially to the observed variability in antioxidant-related parameters. PERMDISP analysis indicated homogeneity of multivariate dispersions among groups (*p* = 0.4063), confirming that observed differences were not due to unequal within-group variability (Table S6).

### 2.3. Phenolic Profile of C. berlandieri subsp. nuttalliae by UPLC-DAD-ESI-QToF/MS

A total of 26 individual phenolic compounds were detected by UPLC-DAD-ESI-QToF/MS analysis (Table 4) and identified based on their exact molecular mass (Table S1). Among the identified compounds, quercetin glucuronide, (iso)rhamnetin glucuronide, quercetin aldopentoside-deoxyhexoside-hexoside, quercetin di-deoxyhexoside-hexoside, (iso)rhamnetin deoxyhexoside-di-hexoside, and quercetin rutinoside, as well as naringin (a flavanone glycoside), showed the highest relative abundance in both plant tissues (Table 4).

Regarding tissue-specific distribution, inflorescences exhibited a higher accumulation of phenolic compounds than leaves. In both tissues, the phenolic profile was predominantly composed of flavonoid derivatives, mainly flavonols derived from quercetin, kaempferol, and isorhamnetin, while naringin was identified as the main flavanone glycoside, representing a distinct subclass within the flavonoid profile and therefore interpreted separately from flavonol-derived compounds. However, differences were observed between tissues in terms of compound concentration, structural complexity, and diversity.

**Figure 2:**
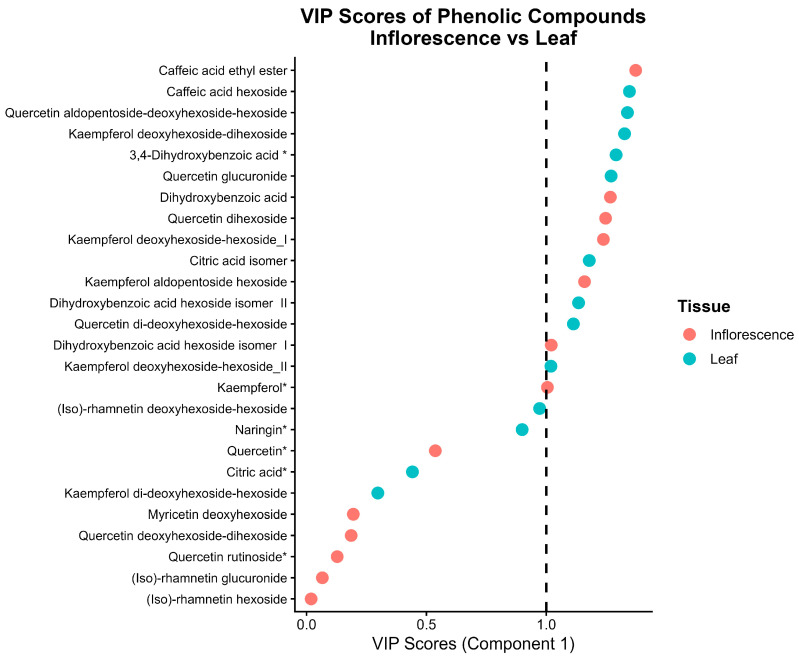
Variable Importance in Projection (VIP) scores of phenolic compounds differentiating inflorescence and leaf tissues of *C. berlandieri* subsp. *nuttalliae*. Each point represents a metabolite identified by UPLC-DAD-ESI-QToF/MS and is colored according to the tissue in which it shows greater discriminatory contribution. VIP scores were obtained from the PLS-DA model (R^2^Y = 0.97, Q^2^ = 0.91, *p* < 0.005, 200 permutations). Compounds with VIP values > 1 were considered relevant contributors to tissue discrimination. The dashed line (VIP = 1) indicates the threshold for relevant metabolites. Compounds marked with an asterisk (*) correspond to phenolic compounds confirmed using reference standards (MSI level 1), whereas the remaining compounds were tentatively identified based on spectral data (MSI level 2), as detailed in Table S1.

Variable Importance in Projection (VIP) scores obtained from the partial least squares discriminant analysis (PLS-DA) model (Figure 2) suggested a differentiated distribution of phenolic compounds contributing to the discrimination between inflorescence and leaf tissues of *C. berlandieri* subsp. *nuttalliae*. In general, phenolic compounds with VIP values > 1 were considered relevant contributors to tissue separation, suggesting differentiated phenolic profiles between both tissues.

In inflorescences, compounds with higher VIP scores included phenolic acids such as *caffeic acid ethyl ester* and *dihydroxybenzoic acid*, as well as glycosylated flavonols including *quercetin dihexoside*, *kaempferol deoxyhexoside-hexoside*, and *kaempferol aldopentoside-hexoside*. Leaves also exhibited compounds with VIP values > 1, including flavonoids, simple phenolic acids, and organic acids. Among the glycosylated flavonoids, *quercetin*, *quercetin dihexoside*, and simple *kaempferol* derivatives were prominent. In addition, phenolic acids and organic acids such as *caffeic acid* and its derivatives, including *protocatechuic acid (3,4-dihydroxybenzoic acid)*, *dihydroxybenzoic acid*, and *citric acid isomers*, contributed to tissue discrimination.

**Figure 3:**
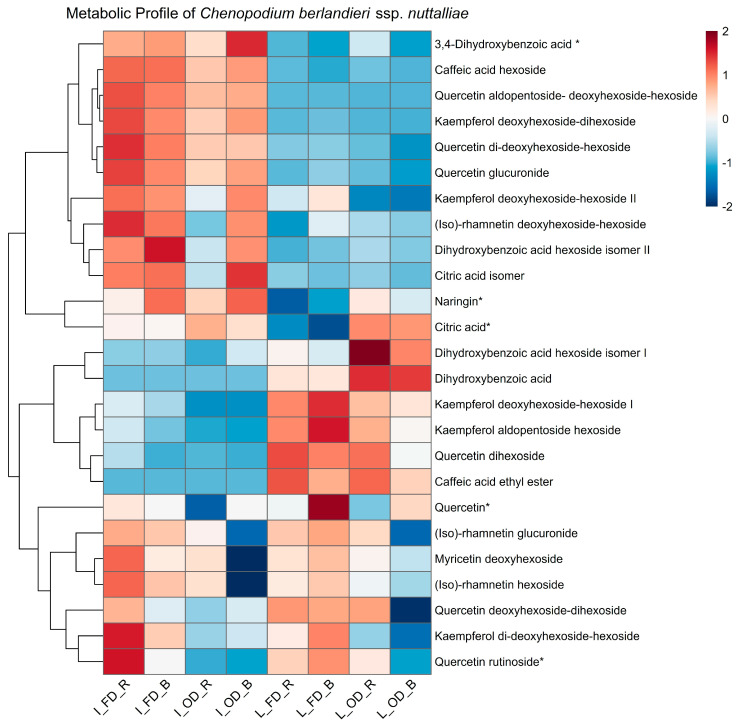
Heatmap illustrating the relative abundance of phenolic acids and flavonoids identified by UPLC-DAD-ESI-QToF/MS in leaves and inflorescences subjected to different drying methods and thermal treatments. Rows correspond to individual phenolic compounds, and columns represent sample groups according to tissue type, drying method, and thermal treatment. Color intensity indicates relative abundance, with higher values shown in red and lower values in blue. Data were normalized by z-score scaling prior to visualization. Hierarchical clustering was performed using Euclidean distance to reveal similarities among samples and metabolites. Compounds marked with an asterisk (*) correspond to phenolic compounds confirmed using reference standards (MSI level 1), whereas the remaining compounds were tentatively identified based on spectral data (MSI level 2), as detailed in Table S1.

**Figure 4:**
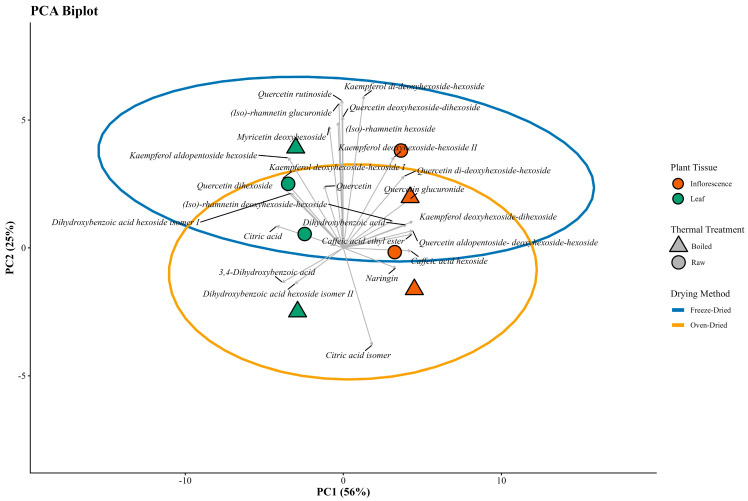
Principal component analysis (PCA) biplot of phenolic compounds identified by UPLC-DAD-ESI-QToF/MS showing sample distribution according to plant tissue, drying method, and thermal treatment. Scores represent individual samples, while vectors indicate the contribution of phenolic compounds to sample separation. Sample color denotes plant tissue (inflorescence and leaf), symbol shape indicates thermal treatment (raw or boiled), and ellipses represent clustering according to drying method (freeze-dried and oven-dried). The percentages on the axes indicate the proportion of total variance explained by each principal component.

The phenolic profile of leaves and inflorescences of *C. berlandieri* subsp. *nuttalliae*, obtained by UPLC-DAD-ESI-QToF/MS analysis (Table 4) and visualized by heatmap representation (Figure 3), showed a clear differentiation according to plant tissue, drying method, and thermal treatment. In inflorescences, complex glycosylated flavonols, including quercetin glucuronide, quercetin aldopentoside-deoxyhexoside-hexoside, and quercetin di-deoxyhexoside-hexoside, exhibited higher relative intensities compared with other phenolic compounds, whereas naringin, identified as a flavanone glycoside, also showed high relative abundance but represents a distinct subclass of flavonoids following a different branch of flavonoid biosynthesis and therefore interpreted separately from flavonol-derived compounds. Freeze-drying was the predominant factor influencing relative abundance, although thermal treatment also contributed to variability in specific compounds. Quercetin glucuronide was the most abundant compound in inflorescences, reaching concentrations of 17,336.48 µg/g in raw freeze-dried samples, whereas raw oven-dried samples showed lower concentrations (13,483.51 µg/g). Similarly, quercetin aldopentoside-deoxyhexoside-hexoside showed concentrations ranging from 4296.74 to 4840.11 µg/g under freeze-drying and 3516.50 to 3758.35 µg/g under oven-drying, while quercetin di-deoxyhexoside-hexoside ranged from 3468.35 to 3816.36 µg/g in freeze-dried samples and 3044.55 to 3069.78 µg/g in oven-dried samples. Some isorhamnetin derivatives were present at higher concentrations in freeze-dried samples, although they were still detectable in certain oven-dried treatments. Overall, these results indicate that complex flavonol glycosides were consistently preserved at higher levels under freeze-drying conditions, particularly in raw samples, suggesting greater stability of these compounds under low-temperature processing. Principal component analysis (PCA) of phenolic metabolite profiles further illustrated the differentiation among samples according to tissue type, drying method, and thermal treatment, as shown in Figure 4.

Regarding thermal treatment, boiling of freeze-dried samples resulted in increased relative abundance of certain compounds, including naringin, quercetin rutinoside, and some isorhamnetin derivatives, compared with raw samples. However, this effect was compound-dependent. For example, quercetin glucuronide decreased from 17,336.48 µg/g in raw freeze-dried samples to 15,726.12 µg/g after boiling. In oven-dried samples, boiling led to reductions in several compounds, such as (iso)rhamnetin deoxyhexoside-hexoside, which decreased from 3138.81 µg/g to 706.45 µg/g after thermal treatment. These findings suggest that thermal treatment induces compound-specific responses, where some flavonoids may increase due to matrix release or transformation, while others are susceptible to degradation depending on the processing conditions. Notably, an increase in kaempferol and its structural derivatives was observed in freeze-dried inflorescences after boiling compared with raw samples. This apparent discrepancy may be explained by the thermal hydrolysis of glycosylated flavonoids, leading to the release of aglycone forms or less complex derivatives that are more readily detected. Additionally, thermal treatment may disrupt matrix–analyte interactions, increasing the extractability of bound flavonols that were not readily detectable in the raw state. This effect may be particularly pronounced in freeze-dried samples due to the porous structure generated during lyophilization, which facilitates solvent penetration and compound release during subsequent thermal processing. Therefore, the observed increase likely reflects transformation and enhanced extractability rather than de novo formation of phenolic compounds.

In leaves, a distinct phenolic profile was observed compared with inflorescences. The heatmap representation (Figure 3) showed a higher contribution of simple flavonoids, phenolic acids, and organic acids in this tissue. According to Table 4, quercetin glucuronide was present at relatively high concentrations in leaves, although at lower levels than those observed in inflorescences. Other compounds with elevated concentrations in leaves included quercetin rutinoside, (iso)rhamnetin glucuronide, myricetin deoxyhexoside, naringin, and phenolic and organic acids such as dihydroxybenzoic acid hexoside isomer II, citric acid, and its isomer.

As shown in the heatmap (Figure 3) and Table 4, the drying method was the main variable associated with changes in the phenolic profile of leaf tissue, whereas the thermal treatment (raw vs. boiled) exerted a secondary effect. Regardless of thermal treatment, freeze-dried samples exhibited higher relative abundance of complex glycosylated flavonols, including (iso)rhamnetin glucuronide, (iso)rhamnetin hexoside, (iso)rhamnetin deoxyhexoside-hexoside, kaempferol deoxyhexoside-hexoside, and quercetin deoxyhexoside-dihexoside, compared with oven-dried samples.

Within the same drying method (freeze-drying), some compounds—such as naringin, (iso)rhamnetin glucuronide, (iso)rhamnetin hexoside, deoxyhexoside-hexoside, and quercetin—showed slight increases in concentration after boiling relative to raw samples, although this effect was not uniform across all compounds.

In contrast, oven-dried samples showed higher concentrations of phenolic acids compared with freeze-dried samples. Compounds such as dihydroxybenzoic acid, dihydroxybenzoic acid hexoside isomers I and II reached their maximum concentrations following thermal treatment. Similarly, organic acids, including citric acid and its isomer, exhibited higher concentrations under oven-drying conditions.

Overall, these patterns indicate a consistent shift from complex flavonol glycosides toward simpler phenolic and organic acids under thermal and oven-drying conditions, highlighting the differential stability and transformation of phenolic compounds depending on processing factors. These patterns should be interpreted as indicative of coordinated metabolite behavior under the evaluated processing conditions, rather than as definitive evidence of biological regulation across independent plant populations.

**Figure 5:**
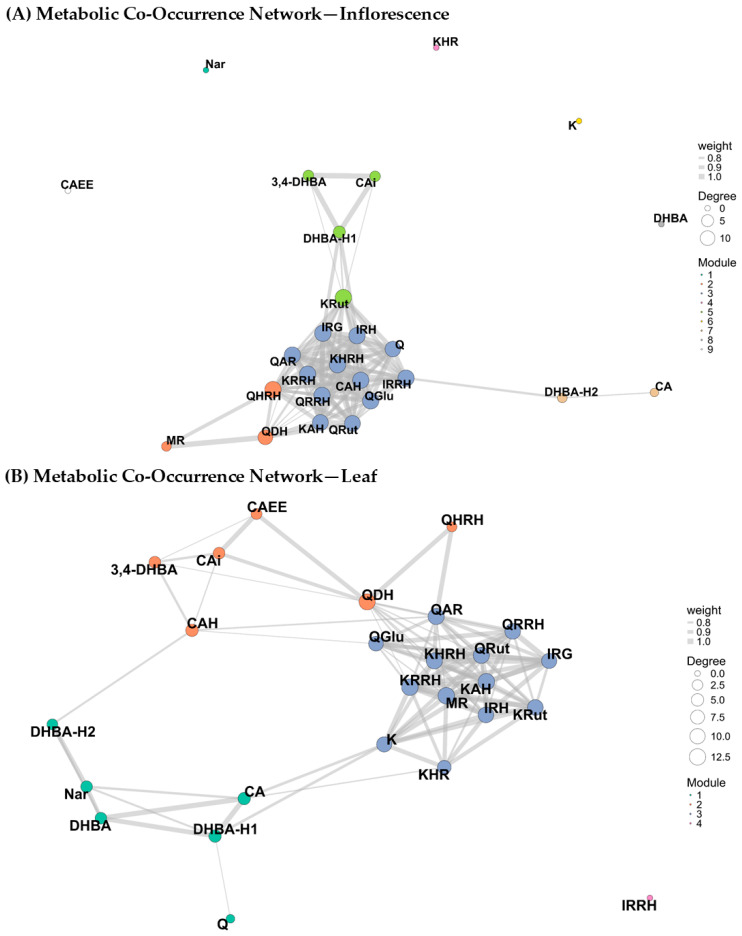
Metabolic co-occurrence networks of phenolic compounds identified in (**A**) inflorescences and (**B**) leaves of *C. berlandieri* subsp. *nuttalliae*. Networks were constructed using significant Pearson correlations (|r| ≥ 0.70, *p* < 0.05) based on log10-transformed metabolomic data. Nodes represent individual metabolites, and node size corresponds to the degree of connectivity. Edge thickness reflects correlation strength (weight). Colors indicate metabolic modules detected using the Louvain community detection algorithm. The networks illustrate the structural organization and connectivity patterns of phenolic metabolites in each plant tissue. Abbreviations: Nar, naringin; QDH, quercetin dihexoside; QHRH, quercetin deoxyhexoside-dihexoside; QRRH, quercetin di-deoxyhexoside-hexoside; QAR, quercetin aldopentoside-deoxyhexoside-hexoside; QRut, quercetin rutinoside; QGlu, quercetin glucuronide; Q, quercetin; KHRH, kaempferol deoxyhexoside-dihexoside; KRRH, kaempferol di-deoxyhexoside-hexoside; KHR, kaempferol deoxyhexoside-hexoside; KAH, kaempferol aldopentoside-hexoside; KRut, kaempferol deoxyhexoside-hexoside; K, kaempferol; IRRH, (iso)-rhamnetin deoxyhexoside-hexoside; IRH, (iso)-rhamnetin hexoside; IRG, (iso)-rhamnetin glucuronide; MR, myricetin deoxyhexoside; DHBA, dihydroxybenzoic acid; DHBA-H1, dihydroxybenzoic acid hexoside isomer I; DHBA-H2, dihydroxybenzoic acid hexoside isomer II; 3,4-DHBA, 3,4-dihydroxybenzoic acid; CAH, caffeic acid hexoside; CAEE, caffeic acid ethyl ester; CA, citric acid; CAi, citric acid isomer.

Metabolomic network analysis revealed the structural organization and topological relevance of phenolic metabolites in both plant tissues of *C. berlandieri* subsp. *nuttalliae* (Figure 5). Differences in network architecture were reflected in connectivity metrics, including degree and betweenness centrality (Table S7).

Although the phenylpropanoid–flavonoid pathway is a well-established biosynthetic route in plants, the present network-based approach provides additional insight into how postharvest processing modulates the co-occurrence structure and connectivity of phenolic compounds. Rather than depicting biosynthetic steps, the metabolic co-occurrence network reflects coordinated variations in compound abundance, which can be interpreted as indirect evidence of shared regulation, transformation processes, or co-extractability patterns under different processing conditions.

The inflorescence network (Figure 5A) showed a central cluster of highly interconnected flavonol derivatives. Flavonol derivatives showed the highest connectivity (degree values approaching the maximum observed in the network), the central hubs correspond mainly to glycosylated derivatives of quercetin, kaempferol, and isorhamnetin. These metabolites formed the central hubs of the phenolic metabolic network, suggesting coordinated regulation among flavonol glycosides. In contrast, hydroxybenzoic acids and simpler phenolic compounds, such as dihydroxybenzoic acid derivatives and citric acid isomers, showed lower connectivity and were mainly located at the periphery of the network. This central organization of flavonol glycosides likely reflects the coordinated regulation of the flavonoid biosynthetic pathway in reproductive tissues. In many plant species, inflorescences accumulate higher levels of flavonol derivatives due to their protective roles against oxidative stress and ultraviolet radiation, as well as their involvement in reproductive development and pollination processes. Therefore, the higher connectivity observed for quercetin-, kaempferol-, and isorhamnetin-derived metabolites suggests that these compounds may represent key intermediates or end-products within the phenylpropanoid–flavonoid metabolic network of *C. berlandieri* subsp. *nuttalliae*.

In contrast, the leaf metabolic network exhibited a more modular and spatially dispersed organization compared with the inflorescence network (Figure 5B). Rather than forming a single highly centralized cluster, the leaf network was structured into several interconnected modules. Flavonol derivatives of quercetin and kaempferol still represented the most connected nodes within the network. Metabolites showing the highest connectivity in leaf tissues (degree ≥ 10) were mainly glycosylated flavonols, including kaempferol aldopentoside hexoside, kaempferol deoxyhexoside-dihexoside, and quercetin rutinoside (Table S7). In contrast, hydroxybenzoic acids, organic acids, and simpler phenolic compounds—such as dihydroxybenzoic acid, caffeic acid ethyl ester, and citric acid—were mainly located in peripheral clusters and displayed lower connectivity within the network.

Consistent with these patterns, the VIP analysis derived from the PLS-DA model indicated that several quercetin- and kaempferol-derived compounds exhibited VIP values greater than one, supporting their contribution to the multivariate discrimination between leaf and inflorescence tissues.

## 3. Discussion

### 3.1. Effect of Drying Methods on the Proximal Composition of Huauzontle

Moisture content is a critical factor influencing the phytochemical stability and microbiological safety of plant-based foods. Lower moisture levels contribute to reduced microbial activity, oxidative reactions, and hydrolytic degradation of antioxidant compounds, thereby favoring longer storage stability [23]. In the present study, moisture content remained below 15% in both leaves and inflorescences regardless of the drying method applied, indicating that both oven-drying and freeze-drying were effective dehydration strategies for *C. berlandieri* subsp. *nuttalliae*. Differences in moisture levels between tissues and drying treatments can be attributed to the distinct dehydration mechanisms involved in each method and to the structural characteristics of the plant matrix. Oven-drying relies on convective heat transfer, promoting the removal of free water from plant tissues, whereas freeze-drying removes frozen water through sublimation under reduced pressure and low temperature. The efficiency of these processes has been reported to depend on tissue architecture and cellular composition, as observed in other plant matrices [15,24].

Ash content, used as an indirect indicator of total mineral content, was higher in leaves than in inflorescences, which is consistent with the physiological roles of foliar tissues in photosynthesis, osmotic regulation, and nutrient transport [25]. Although ash values obtained in this study were lower than those reported for *C. berlandieri* subsp. *berlandieri* and other *quelite* species such as romeritos and verdolagas [10,15], they were higher than those reported for *Porophyllum ruderale* [26], highlighting interspecific and tissue-dependent variability in mineral accumulation. Although ash content represents inorganic mineral components that are not chemically altered by drying processes, the differences observed among treatments may be attributed to changes in concentration on a dry weight basis and to matrix-related effects that influence moisture removal efficiency. Therefore, variations in ash content are likely associated with differences in water loss and sample composition rather than actual changes in mineral content.

Protein content in *C. berlandieri* subsp. *nuttalliae* was notably high and remained stable across drying treatments, supporting previous observations that dehydration processes do not significantly affect protein levels in studies on *quelites* [15,27]. The protein values observed in this study were comparable to those reported for other nutritionally relevant *quelites* such as quintonil and verdolaga [28], and considerably higher than those described for various edible and medicinal plants [29], reinforcing the nutritional potential of huauzontle as a plant-based protein source.

Lipid content was significantly higher in inflorescences than in leaves, which may be related to the presence of a diverse array of lipid classes, including fatty acids, phospholipids, galactolipids, sphingolipids, sterols, and neutral lipids, associated with structural functions and carbon and energy storage during reproductive development [30]. The higher lipid levels observed in inflorescences compared to *C. berlandieri* subsp. *berlandieri* suggest that reproductive tissues of huauzontle may represent an underexplored source of plant lipids with potential biological relevance [29].

Dietary fiber content in leaves was lower than that reported for several *quelite* species, including huauzontle, quintonil, and verdolaga, as well as for *C. berlandieri* subsp. *berlandieri* [10,15]. Dietary fiber consists mainly of structural polysaccharides such as cellulose, hemicelluloses, and pectins, which are associated with colonic fermentation, short-chain fatty acid production, and modulation of glucose and lipid metabolism, as well as anti-inflammatory and prebiotic effects [31]. Differences in fiber content among *quelites* may reflect species-specific cell wall composition and tissue structure.

Finally, the total carbohydrate content in *C. berlandieri* subsp. *nuttalliae* was comparable to that reported for romeritos but lower than values reported for huauzontle, quintonil, and verdolaga in previous studies [10]. Carbohydrates in plants include both structural and non-structural fractions, which play essential roles in energy storage, metabolic support, and reproductive development [32,33]. From a nutritional perspective, carbohydrate- and fiber-rich plant foods contribute to energy balance, glycemic control, lipid metabolism, and gut fermentation in humans [34], underscoring the nutritional relevance of huauzontle within traditional diets.

### 3.2. Effect of Drying and Thermal Treatment on Phenolic Composition and Antioxidant Capacity

The phenolic composition and antioxidant capacity of *C. berlandieri* subsp. *nuttalliae* were strongly influenced by both the drying method and thermal treatment, with clear tissue-dependent responses. Overall, freeze-drying favored the preservation of phenolic compounds and antioxidant activity, particularly in inflorescences, whereas oven-drying and boiling were associated with variable reductions depending on the compound class and plant tissue. These findings are consistent with previous studies in other plant matrices, where freeze-drying has been shown to better preserve phenolic compounds and antioxidant capacity compared to conventional thermal drying methods [35,36,37]. This effect has been attributed to the low-temperature conditions of freeze-drying, which limit enzymatic degradation and oxidative reactions, thereby maintaining the structural integrity of phenolic compounds. In contrast, thermal drying methods may promote degradation, transformation, or loss of these compounds, resulting in reduced antioxidant capacity.

Total phenolic content was maximized in raw freeze-dried inflorescences, indicating that low-temperature dehydration effectively limits thermal degradation and enzymatic oxidation of phenolic compounds. In contrast, lower TPC values observed in oven-dried and boiled samples suggest the occurrence of heat-induced degradation, potential involvement of Maillard-type reactions with pro-oxidant effects, and leaching of water-soluble phenolics during boiling [38,39]. Interestingly, leaf tissues showed a different response, with raw oven-dried leaves exhibiting relatively high TPC values, highlighting tissue-specific differences in phenolic localization, matrix composition, and thermal sensitivity.

Flavonoid content showed greater susceptibility to combined thermal stress, particularly in oven-dried and boiled samples. This reduction may be attributed to structural modifications of the plant cell wall and enhanced oxidation during exposure to elevated temperatures and aqueous environments [40]. In addition, most flavonoids in vegetables occur as glycosylated derivatives, which can undergo hydrolysis or degradation under prolonged thermal conditions, along with losses due to lixiviation of hydrophilic compounds [39,41]. The relatively stable TFC observed in freeze-dried boiled inflorescences suggests that freeze-drying may partially protect flavonoid structures against subsequent thermal processing.

Condensed tannins exhibited marked variability among treatments, reflecting their sensitivity to both drying conditions and boiling. Although freeze-drying has been reported to preserve the physicochemical quality of plant matrices in some species [42], the present results indicate that CT responses are highly dependent on tissue type and processing sequence. The observed reduction in CT following the combination of thermal drying and boiling is consistent with previous reports describing the destabilization of high-molecular-weight tannins under prolonged heat exposure [43,44].

Antioxidant capacity assessed by DPPH, ABTS, and FRAP assays followed trends largely consistent with total phenolic content, reinforcing the well-established relationship between phenolic compounds and antioxidant activity through electron donation and reducing power mechanisms [38]. Among the evaluated variables, total phenolic content (TPC) appears to be the primary factor associated with antioxidant capacity (DPPH, ABTS, and FRAP), showing the most consistent relationship across treatments. In contrast, total flavonoid content (TFC) exhibited a less consistent pattern, suggesting a secondary contribution. This behavior is consistent with multivariate analyses, where TPC and antioxidant-related variables were closely associated, reinforcing the role of phenolic compounds as the main determinants of antioxidant capacity. Raw inflorescences, particularly when freeze-dried, consistently exhibited higher antioxidant capacity across all assays, whereas boiling led to significant reductions in most cases. Notably, freeze-dried leaves displayed increased ABTS values after boiling, suggesting that thermal treatment may enhance the extractability of certain bound phenolics in specific tissues.

Multivariate analyses provided an integrated view of these effects. PCA clearly demonstrated that the drying method was the primary driver of variability in both inflorescences and leaves, with freeze-dried samples clustering according to higher TPC and antioxidant capacity, whereas oven-dried samples were associated with higher CT and DPPH-related responses. The secondary separation driven by thermal treatment highlights the additional influence of boiling on phenolic stability. PERMANOVA results further supported that tissue type, drying method, and thermal treatment—and particularly their interactions—significantly shaped the overall phenolic and antioxidant profiles, underscoring the complex and tissue-specific nature of processing effects.

Collectively, these findings demonstrate that freeze-drying is the most effective strategy for preserving the phenolic integrity and antioxidant potential of *C. berlandieri* subsp. *nuttalliae*, especially in inflorescences, while thermal processing introduces compound- and tissue-dependent modifications. This integrated response highlights the importance of considering both postharvest and culinary processing when evaluating the functional potential of traditional edible plants such as huauzontle.

### 3.3. Individual Phenolic Profile by UPLC-DAD-ESI-QToF/MS

The higher accumulation of phenolic compounds observed in inflorescences compared with leaves indicates a clear tissue-dependent specialization of phenolic metabolism in *C. berlandieri* subsp. *nuttalliae*, a pattern that has also been reported in other quelite species [15]. Although both tissues shared a phenolic profile dominated by flavonol derivatives of quercetin, kaempferol, and isorhamnetin, the inflorescences exhibited greater diversity and structural complexity of flavonoid conjugates [45,46]. This differentiation suggests a coordinated regulation of phenolic biosynthesis associated with tissue function and developmental stage. In many plant species, reproductive tissues accumulate high levels of flavonoids, which contribute to protection against ultraviolet radiation and play an important role in the antioxidant defense system of the plant, as reported by Agati et al. [47].

The predominance of glycosylated flavonols in both tissues is consistent with the role of flavonoid conjugation in modulating solubility, stability, and compartmentalization within plant tissues [46,48]. Differences in relative abundance and diversity among tissues further support the tissue-specific phenolic signatures observed in the heatmap and multivariate analyses [49] (Figure 3 and Figure 5), as well as in the correlation-based metabolic network (Figure 5).

The PLS-DA model highlighted a clear tissue-dependent contribution of specific phenolic compounds, as reflected by VIP scores greater than one. The predominance of phenolic acids and structurally complex glycosylated flavonols in inflorescences suggests a specialized phenolic metabolism potentially associated with reproductive tissue function [15]. In contrast, leaves were characterized by a broader contribution of simple phenolic acids, organic acids, and less complex flavonoid derivatives, indicating a distinct metabolic strategy linked to vegetative tissues [50].

The identification of compounds such as caffeic acid derivatives, dihydroxybenzoic acids, and citric acid isomers as relevant discriminators in leaves points to metabolic pathways related to primary–secondary metabolism crosstalk and stress-related functions [51]. Conversely, the enrichment of highly glycosylated flavonols in inflorescences supports their role in pigment stabilization, antioxidant defense, and protection of reproductive structures [52,53]. These tissue-specific VIP patterns are consistent with the clustering observed in the PCA and heatmap analyses (Figure 3 and Figure 5) and with the modular organization of metabolites revealed by the correlation-based metabolic network (Figure 5).

The highly interconnected network observed in inflorescences reflects a coordinated regulation of phenolic metabolism dominated by structurally complex glycosylated flavonols [50]. The presence of modules enriched in glycosylated derivatives of quercetin, kaempferol, and isorhamnetin with high degree values suggests that these metabolites constitute the central axis of the inflorescence phenolic profile, potentially related to protective and regulatory functions in reproductive tissues (Figure 5; Table S7) [46,53].

In contrast, the module composed mainly of hydroxybenzoic acids and simple phenolic compounds exhibited lower connectivity, indicating a more peripheral role within the network. Metabolites showing high betweenness centrality but low degree may act as metabolic bridges connecting different subnetworks, thereby facilitating coordination between phenolic subpathways [45,50]. This topological behavior supports the concept of hierarchical organization within phenolic metabolism, where highly connected flavonol conjugates dominate network structure, while simpler phenolic acids contribute to metabolic flexibility and inter-module communication [50,51].

The high centrality and accumulation of phenolic metabolites observed in inflorescences indicate a coordinated metabolic regulation of this tissue, which is further supported by the VIP analysis derived from the PLS-DA model (Figure 2). Flavonol derivatives of quercetin and kaempferol exhibited VIP values greater than one, confirming their major contribution to multivariate separation between tissues [53].

Metabolites such as quercetin deoxyhexoside-dihexoside, quercetin dihexoside, and kaempferol deoxyhexoside-hexoside showed both high network connectivity and elevated VIP scores, suggesting that these compounds act as key discriminant markers and may function as metabolic bridges linking different modules within the phenolic network [50]. This dual relevance highlights their central role in structuring the phenolic profile of inflorescences.

In contrast, phenolic acids such as caffeic acid ethyl ester, dihydroxybenzoic acid, and citric acid isomers exhibited lower connectivity within the metabolic network but still showed VIP values greater than one. This pattern indicates that, although these metabolites do not act as central hubs in the network, their concentrations differ significantly between tissues and contribute to tissue discrimination primarily through differences in abundance rather than network centrality [45,50]. Additionally, some metabolites, such as naringin and kaempferol, appeared as isolated nodes (degree = 0), indicating the absence of significant correlations with other metabolites under the applied network threshold (Table S7).

The more dispersed and modular organization observed in the leaf metabolic network suggests a phenolic metabolism structured around fewer highly connected flavonol derivatives, primarily quercetin- and kaempferol-based conjugates [50]. These metabolites appear to constitute the core of the leaf phenolic network, in contrast to the more densely interconnected structure observed in inflorescences (Figure 5; Table S7).

The lower connectivity of hydroxybenzoic acids, organic acids, and simple phenolic compounds indicates a peripheral role within the network, despite their contribution to tissue discrimination as revealed by VIP analysis. This pattern suggests that, in leaves, multivariate separation is driven by differences in metabolite abundance rather than by centrality within the metabolic network [50]. Together, the combined interpretation of network topology and PLS-DA results highlights a tissue-specific organization of phenolic metabolism, with distinct structural and discriminatory roles for individual compounds in leaves and inflorescences [4,15].

Network topology analysis further revealed that certain flavonol derivatives act as metabolic bridges linking different phenolic modules. In particular, quercetin dihexoside exhibited a relatively high betweenness centrality value (0.19), suggesting a potential role in connecting highly glycosylated flavonols with less glycosylated phenolic compounds within the metabolic network (Table S7). This topological position indicates that quercetin dihexoside may function as a connector metabolite linking distinct phenolic subpathways. Such connector metabolites may contribute to the coordination of flavonol glycosylation patterns within the phenylpropanoid pathway.

Similarly, caffeic acid hexoside and several kaempferol derivatives exhibited elevated betweenness centrality values together with high VIP scores, supporting their dual role as inter-module connectors and discriminant metabolites, particularly in leaf tissues. The convergence of high network centrality and multivariate importance highlights these compounds as both structurally and statistically relevant contributors to tissue-specific phenolic differentiation. A fourth module composed of a single node was also observed, corresponding to (iso)-rhamnetin deoxyhexoside-hexoside. This bioactive compound showed no significant correlations with other metabolites (degree = 0), indicating an independent behavior within the phenolic profile of leaf tissue (Table S7) [4,54].

In contrast, metabolites such as dihydroxybenzoic acid, caffeic acid ethyl ester, and citric acid exhibited significant VIP values but a low degree within the metabolic network [50,54]. This pattern indicates that their contribution to tissue discrimination is primarily driven by quantitative differences in concentration rather than by a central structural role within the phenolic network (Table S7) [45]. Together, these findings reinforce the complementary value of integrating network topology and multivariate analyses to elucidate tissue-specific organization and regulation of phenolic metabolism [45,50,51].

The heatmap visualization and quantitative data indicate a strong influence of drying method on the preservation and relative abundance of individual phenolic compounds, particularly in inflorescences [15]. The consistently higher concentrations of complex glycosylated flavonols under freeze-drying, along with the absence of several compounds in oven-dried samples, suggest that low-temperature dehydration limits degradation or loss of thermolabile phenolics [15,46]. The compound-dependent response to thermal treatment further highlights the complexity of phenolic stability [15,53]. While thermal processing may enhance the extractability of certain flavonoids in some cases, this effect was both compound- and matrix-dependent. While some flavonoids showed slight increases in freeze-dried samples, others, such as quercetin glucuronide, decreased following the thermal treatment. In oven-dried samples, boiling generally intensified compound losses, suggesting a cumulative effect of thermal exposure during both drying and cooking processes [4,15].

Overall, these patterns support the notion that freeze-drying provides better preservation of structurally complex flavonol conjugates, whereas oven-drying combined with boiling may exacerbate phenolic degradation. The differential behavior among compounds underscores the importance of considering both postharvest processing and culinary treatment when evaluating the phenolic composition of *C. berlandieri* subsp. *nuttalliae* [4,15]. The contrasting phenolic profiles observed between leaves and inflorescences reflect tissue-specific responses to postharvest processing and thermal treatment [45,50]. In leaves, the predominance of simple flavonoids, phenolic acids, and organic acids suggests a phenolic metabolism oriented toward compounds with lower structural complexity, in contrast to the inflorescence profile enriched in highly glycosylated flavonols [15,46,55]. The heatmap analysis highlights the drying method as the primary driver of phenolic variation in leaf tissue, while thermal treatment plays a secondary role. The consistent enrichment of complex flavonol conjugates in freeze-dried samples supports the notion that low-temperature dehydration favors the preservation of thermolabile and highly glycosylated phenolics [15,56]. Conversely, the higher abundance of phenolic and organic acids in oven-dried samples—particularly following boiling—suggests enhanced degradation of complex flavonoids or increased release of simpler phenolic structures under cumulative thermal exposure [4,15].

The compound-dependent response to boiling observed in freeze-dried leaves further emphasizes the dynamic nature of phenolic stability, where thermal treatment may enhance extractability of certain metabolites while reducing others [4,15,46]. Together, these findings reinforce the importance of considering both tissue type and processing conditions when evaluating the phenolic composition and functional potential of *C. berlandieri* subsp. *nuttalliae* [4,15]. The correlation-based metabolic network exhibited a modular organization in which modules enriched in flavonoid derivatives were mainly associated with inflorescence tissues. In contrast, other modules grouped metabolites such as phenolic acids and simpler flavonoid derivatives that were primarily detected in leaf samples [45,50]. This modular distribution suggests tissue-specific regulation of phenylpropanoid metabolism, where reproductive tissues tend to accumulate structurally complex flavonol glycosides, while vegetative tissues display a broader distribution of simpler phenolic metabolites. Such tissue-related modularity indicates coordinated regulation of phenolic metabolism and highlights distinct phenolic signatures in *C. berlandieri* subsp. *Nuttalliae.*

A limitation of this study is the use of plant material obtained from a local market, which may introduce variability related to origin, handling, and physiological stage. However, the traceability of the material was strengthened by verifying its origin (Atlixco, Puebla, Mexico) and by processing successive batches (~10 kg fresh biomass per batch) to generate homogenized composite samples (12 kg dry matter in total), thereby improving representativeness and reducing variability associated with market sourcing. Although taxonomic identity was verified and samples were selected based on uniform morphological characteristics, uncontrolled pre-harvest and postharvest factors cannot be entirely excluded. Thus, the results should be interpreted as representative of commonly consumed plant material under real-world conditions rather than controlled agronomic systems. Furthermore, all experimental treatments were applied to the same homogenized composite material, ensuring internal consistency and allowing valid comparative assessment of drying and thermal processing effects. In addition, the study was based on analytical replicates derived from homogenized composite samples rather than independent biological replicates. While the use of composite samples improved representativeness and reduced within-sample variability, it does not capture biological variability across independent plant sources. Consequently, PCA and PLS-DA results should be interpreted as exploratory and indicative of general trends rather than definitive inferential evidence. Future studies incorporating independent biological replicates and controlled cultivation conditions are required to confirm these observations.

## 4. Materials and Methods

### 4.1. Plant Material

The plant material was taxonomically validated using the World Flora Online database (https://worldfloraonline.org/taxon/wfo-0000601083; accessed on 1 December 2025). Leaves and inflorescences of *C. berlandieri* subsp. *nuttalliae* were obtained from the Escobedo market in the state of Querétaro, Mexico, during March and April 2024. According to information provided by the supplier and verified through direct inquiry at the point of purchase, the plant material originated from Atlixco, Puebla, Mexico (18°55′46.2″ N, 98°26′11.5″ W), one of the main commercial production regions of huauzontle in the country. This region is characterized by a temperate subhumid climate (Cwb), with an average annual temperature of approximately 18–19 °C, mean daily temperatures around 17–18 °C, and typical ranges from ~8–14 °C (minimum) to ~25–28 °C (maximum) depending on the season. During the production and collection period (March–April), average temperatures range from approximately 13–20 °C, with maximum values near 27–28 °C and minimum values around 11–13 °C. These mild thermal conditions, combined with seasonal rainfall patterns, are considered favorable for the cultivation of traditional leafy vegetables such as huauzontle. Puebla is recognized as the principal producer of huauzontle in Mexico, contributing more than 90% of the national production.

The specimens were identified by a specialist at the Jerzy Rzedowski Herbarium and deposited as a voucher specimen under accession number QMEX00007774 at the Universidad Autónoma de Querétaro. Plant material was acquired in successive batches of approximately 10 kg of fresh biomass, which were selected and processed under consistent laboratory conditions. This batch-based sampling approach allowed the generation of representative composite samples used throughout the study.

To minimize variability associated with market sourcing, samples were carefully selected based on uniform morphological characteristics, including size, color, and apparent maturity stage. Additionally, all samples were processed under identical experimental conditions to ensure consistency across treatments. The use of plant material obtained from local markets is representative of real consumption patterns of traditional edible plants (quelites) in the Mexican population, thereby increasing the ecological relevance of the study.

### 4.2. Drying and Extraction Procedures

After collection, the plant material was cleaned, and the main stem, which is typically not consumed, was removed and discarded. Leaves and inflorescences were then separated manually. The proportion between plant tissues was not uniform, with inflorescences representing approximately 90% of the biomass and leaves about 10%. Leaves and inflorescences were subsequently subjected to two drying methods: (1) oven-drying with forced air circulation at 40 °C Shel-Lab FX1375 (Sheldon Manufacturing Inc., Cornelius, OR, USA) until constant weight was achieved, and (2) freeze-drying at −55 °C and 1 Pa using a laboratory freeze dryer (10 N Series, SCIENTZ, Ningbo, Zhejiang, China). The plant material was processed in successive batches of approximately 10 kg of fresh biomass, which were handled under the same laboratory conditions. This procedure was repeated until a total of 12 kg of dry matter was obtained, distributed into four composite samples: 3 kg of oven-dried leaves, 3 kg of freeze-dried leaves, 3 kg of oven-dried inflorescences, and 3 kg of freeze-dried inflorescences. Each composite sample was thoroughly homogenized to ensure consistency prior to further processing. Dried samples were ground and sieved to a particle size of 0.5 mm, and the resulting powders were stored in airtight bags at −80 °C until further processing. After drying, the samples were divided into two groups: raw and boiled. For both treatments, all subsamples were taken from the corresponding homogenized composite material, ensuring that raw and boiled treatments originated from the same processed biomass. For the boiled treatment, 5 g of dried plant material were mixed with 100 mL of distilled water and heated at 100 °C for 5 min. The samples were then cooled to room temperature, and the volume loss was adjusted back to 100 mL with distilled water. Subsequently, 400 mL of absolute methanol were added to obtain a hydroalcoholic solution (80:20, *v*/*v*, methanol:water), which was used for extraction. For the raw treatment, 5 g of dried powdered sample were directly mixed with 500 mL of a methanol–water solution (80:20, *v*/*v*), corresponding to a solid-to-liquid ratio of 1:100 (g/mL). Extraction was performed by magnetic stirring OM10E oven (OVAN, Burladingen, Germany) at 100 rpm and 22 ± 1 °C for 16 h in the absence of light. The extracts were then filtered through Whatman No. 541 filter paper and concentrated under reduced pressure using a rotary evaporator (R-200, Büchi, Flawil, Switzerland) at 40 °C and 100 mmHg. The concentrated extracts were subsequently freeze-dried and stored at −80 °C until analysis.

### 4.3. Proximal Chemical Analysis

Proximal chemical analysis of the plant material was performed according to official methods of the Association of Official Analytical Chemists (AOAC) [57]. The analysis was performed using dried plant material obtained from fresh (non-boiled) samples that were previously subjected to oven-drying or freeze-drying treatments as part of the experimental design. No thermal cooking treatments (e.g., boiling) were applied prior to proximal analysis. The following parameters were determined: moisture content (method 925.10), ash content (method 942.05), protein content (method 920.87), lipid content (method 920.39), and dietary fiber (method 962.09).

Total carbohydrate content was calculated by difference using the following equation:

$$\mathrm{Total}\;\mathrm{carbohydrates}\;(\%)=100-\left[\%\left(\mathrm{moisture}+\mathrm{dietary}\;\mathrm{fiber}+\mathrm{lipids}+\mathrm{ash}+\mathrm{protein}\right)\right]$$

All analyses were performed in triplicate for each treatment (*n* = 3), and results were expressed as percentage (%) on a dry matter basis.

### 4.4. Determination of Total Phenolic Content, Total Flavonoid Content, and Condensed Tannins

Total phenolic content (TPC) was determined using the Folin–Ciocalteu colorimetric method as described by Singleton et al. [58]. Results were expressed as milligrams of gallic acid equivalents (mg GAE/g DE). Total flavonoid content (TFC) was determined according to the colorimetric method reported by Zhishen et al. [59], and results were expressed as milligrams of (+)-catechin equivalents (mg CE/g DE). Condensed tannins (CT) were quantified using the spectrophotometric method described by Feregrino-Pérez et al. [60], with results also expressed as milligrams of catechin equivalents (mg CE/g DE).

### 4.5. Antioxidant Capacity

Antioxidant capacity was evaluated using three spectrophotometric assays. The DPPH radical scavenging assay (1,1-diphenyl-2-picrylhydrazyl) was performed according to the method described by Brand-Williams et al. [61]. The ABTS radical cation decolorization assay [2,2′-azino-bis(3-ethylbenzothiazoline)-6-sulfonic acid] was carried out following the procedure reported by Ozgen et al. [62]. The ferric reducing antioxidant power (FRAP) assay was conducted as described by Benzie and Strain [63]. Results from all three antioxidant assays were expressed as micromoles of Trolox equivalents per gram of dried extract (µmol TE/g DE).

### 4.6. Identification and Quantification of Individual Phenolic Compounds by UPLC-DAD-ESI-QToF/MS

Individual phenolic compounds were identified and quantified using ultra-performance liquid chromatography (UPLC) coupled to diode array detection (DAD) and quadrupole time-of-flight mass spectrometry with electrospray ionization (ESI-QToF/MS) (Waters Corporation, Milford, MA, USA), following the methodology reported by Reynoso-Camacho et al. [64], with minor adaptations. Prior to injection, 25 mg of dried extract were resuspended in 500 µL of MS-grade water, and 2 µL of each sample were injected into the system. Chromatographic separation was performed on an Acquity BEH C18 column (2.1 × 100 mm, 1.7 µm particle size; Waters Corp., Milford, MA, USA) maintained at 35 °C. The mobile phase consisted of water with 0.1% formic acid (A) and 100% acetonitrile (B), both of MS grade. The gradient elution program was as follows at a flow rate of 0.5 mL/min: 0% B (0 min), 15% B (2.5 min), 21% B (10 min), 90% B (12 min), 95% B (13 min), and 0% B (15 min). UV–Vis absorbance spectra were recorded at 214, 280, 320, and 360 nm. Quantification of individual phenolic compounds was carried out using external calibration curves prepared with HPLC-grade commercial standards, including naringin, quercetin, rutin (quercetin rutinoside), kaempferol, 3,4-dihydroxybenzoic acid, caffeic acid, and citric acid. Compounds lacking corresponding standards were semi-quantified and expressed as equivalents of structurally related compounds within the same subclass (Table S1). The calibration curves equations and validation parameters are included in the Supplementary Material (Table S2). Calibration curves showed high linearity (R^2^ > 0.98), and LOD and LOQ values were determined for each standard. Quantitative results were expressed as micrograms of each phenolic compound per gram of dried extract (µg/g DE).

Mass spectrometric analysis was performed in negative ionization mode (ESI−) under the following conditions: capillary voltage, 2.0 kV; cone voltage, 40 V; low collision energy, 6 V; high collision energy, 15–45 V; source temperature, 120 °C; cone gas flow, 50 L/h; and desolvation gas flow (N_2_), 800 L/h at 450 °C. Data acquisition was carried out over an *m*/*z* range of 100–1200, with a mass resolution of 35,000 FWHM (*m*/*z* 556.2771, ESI− mode). Mass calibration was performed using leucine enkephalin as lock mass (50 pg/mL; 10 µL/min). Compounds for which authentic commercial standards were available were confirmed (level 1) based on retention time, exact mass (mass error < 5 ppm), and MS/MS fragmentation patterns. For the remaining compounds, identification was considered putative (level 2) and was based on exact mass measurements (mass error < 5 ppm), isotopic distribution, MS/MS fragmentation patterns, and comparison with databases and previously reported phenolic compounds. Detailed identification parameters are provided in the Supplementary Material (Table S1). For putatively identified compounds, structural annotation was limited to the aglycone and general conjugation type; however, the exact identity, linkage position, and stereochemistry of sugar moieties could not be determined in the absence of authentic standards. Representative MS/MS spectra supporting compound identification are provided in the Supplementary Figures S2–S6.

### 4.7. Statistical and Multivariate Analysis

All statistical analyses were performed using R software (v4.4.1). Results for total phenolic content (TPC), total flavonoid content (TFC), condensed tannins (CT), and antioxidant capacity (DPPH, FRAP, and ABTS) were expressed as mean ± standard deviation (*n* = 9, corresponding to analytical replicates for each treatment). Differences among treatments were evaluated using one-way analysis of variance (ANOVA) followed by Tukey’s post hoc test (*p* < 0.05). Prior to multivariate analyses, variables were mean-centered and scaled to unit variance (z-scores). Principal component analysis (PCA) was performed separately for leaves and inflorescences to reduce tissue-driven variability and to evaluate the combined effects of drying method and thermal treatment on phenolic composition and antioxidant capacity. For PCA, mean values of each experimental treatment were used, resulting in eight observations corresponding to the combination of tissue type (inflorescence and leaf), drying method (freeze-drying and oven-drying), and thermal treatment (raw and boiled). Because multivariate analyses were performed using mean values of analytical replicates rather than independent biological replicates, PCA was used as an exploratory tool for pattern recognition and visualization of treatment-related trends. Multivariate differences among tissue type, drying method (freeze-drying vs. oven-drying), thermal treatment (raw vs. boiled), and their interactions were assessed using permutational multivariate analysis of variance (PERMANOVA) based on Euclidean distances with 999 permutations. Homogeneity of multivariate dispersions was verified using PERMDISP (999 permutations) (Table S6). Metabolomic data obtained from UPLC-DAD-ESI-QToF/MS were autoscaled prior to supervised modeling using partial least squares discriminant analysis (PLS-DA) to discriminate between tissues. Model performance was evaluated using R^2^ and Q^2^ parameters obtained through internal cross-validation. Similarly, PLS-DA was applied as an exploratory supervised approach to support pattern discrimination, and its results were interpreted with caution given the absence of biological replication. Discriminant metabolites were identified based on variable importance in projection (VIP) scores > 1.0. VIP-based interpretation was complemented with PCA, heatmap visualization, and network analysis to avoid overinterpretation of individual variables. Metabolic co-occurrence networks were constructed using Spearman correlation coefficients (|ρ| ≥ 0.70; *p* ≤ 0.05) calculated from the metabolite abundance matrix. Topological network parameters, including degree and betweenness centrality, were subsequently computed, and community structure was identified using the Louvain community detection algorithm.

## 5. Conclusions

The results of this study suggest that both drying method and thermal treatment significantly influence the phenolic profile and antioxidant capacity of *C. berlandieri* subsp. *nuttalliae* leaves and inflorescences. In contrast, the nutritional composition of both tissues remained largely stable, as carbohydrate, protein, and dietary fiber contents were not significantly affected by the drying processes, indicating that post-harvest dehydration may not substantially alter the macronutrient profile of this plant.

Freeze-drying appeared to be the most effective method for preserving bioactive compounds and antioxidant capacity, particularly complex glycosylated flavonols such as quercetin glucuronide. In inflorescences, freeze-dried samples showed reductions in certain metabolites after boiling, whereas oven-dried inflorescences exhibited marked losses or complete absence of some (iso)rhamnetin- and myricetin-derived compounds. Conversely, the concentration of other phenolic compounds increased after boiling, indicating a metabolite-dependent response to thermal treatment.

Leaf tissues exhibited a distinct behavior compared with inflorescences. Freeze-dried leaves showed increased concentrations of several flavonoid compounds after boiling, which may be associated with enhanced release of phenolics bound to the plant matrix. In contrast, oven-dried leaves subjected to boiling showed a pronounced decrease in phenolic compounds, with higher concentrations retained in raw samples.

Overall, these findings suggest that both tissue type and processing conditions play an important role in determining the phenolic composition and antioxidant potential of *C. berlandieri* subsp. *nuttalliae*. The consumption of this traditional quelite in dried form may represent a valuable source of nutrients and bioactive compounds with antioxidant capacity, supporting its potential contribution to human health. However, these results should be interpreted within the context of the experimental conditions evaluated, and further studies under controlled conditions are required to confirm these findings. Nevertheless, further in vitro and in vivo studies are required to evaluate the bioaccessibility, bioavailability, and biological effects of the phenolic compounds present in *C. berlandieri* subsp. *nuttalliae*.

## Figures and Tables

**Table 1:** Proximal chemical composition of leaves and inflorescences of *C. berlandieri* subsp. *nuttalliae* subjected to different drying methods and thermal treatments. Analyses were performed on dried samples obtained from fresh (non-boiled) plant material.

	Freeze-Dried Inflorescence	Oven-Dried Inflorescence	Freeze-Dried Leaf	Oven-Dried Leaf
Moisture (%)	4.89 ± 0.02 ^a^	3.15 ± 0.02 ^b^	4.67 ± 0.03 ^c^	4.76 ± 0.00 ^d^
Ash (%)	11.69 ± 0.07 ^a^	12.77 ± 0.07 ^b^	13.99 ± 0.08 ^c^	13.37 ± 0.11 ^d^
Protein (%)	27.76 ±0.31 ^a^	28.32 ± 0.33 ^ab^	27.43 ± 0.03 ^a^	28.77 ± 0.15 ^b^
Lipid (%)	3.59 ± 0.17 ^a^	3.56 ± 0.07 ^a^	2.75 ± 0.02 ^b^	2.88 ± 0.08 ^b^
Dietary fiber (%)	7.32 ± 0.69 ^a^	7.16 ± 0.88 ^a^	6.96 ± 1.01 ^a^	6.85 ± 0.72 ^a^
Carbohydrates (%)	44.76 ± 0.30 ^a^	45.04 ± 0.43 ^a^	44.21 ± 0.86 ^a^	43.37 ± 0.68 ^a^

Values are expressed as mean ± standard deviation (*n* = 9). Different lowercase letters (a–d) within each row indicate significant differences among drying and thermal treatments (one-way ANOVA, Tukey’s post hoc test, *p* < 0.05).

**Table 2:** Total phenolics, flavonoids, tannins and antioxidant capacities in huauzontle (*C. berlandieri* ssp. *nuttalliae*) tissues subjected to oven- and freeze-drying.

Sample	TPC (mg GAE/g DE)	TFC (mg CE/g DE)	CT (mg CE/g DE)	DPPH (µM TE/g DE)	ABTS (µM TE/g DE)	FRAP (µM TE/g DE)
I_OD_R	22.89 ± 1.19 ^d^	12.83 ± 0.59 ^a^	1.08 ± 0.06 ^a^	566.48 ± 27.16 ^a^	564.35 ± 17.54 ^c^	239.05 ± 18.51 ^c^
I_OD_B	17.98 ± 1.89 ^e^	6.87 ± 0.48 ^e^	0.84 ± 0.05 ^b^	500.19 ± 27.39 ^b^	509.53 ± 12.18 ^e^	144.75 ± 5.84 ^e^
I_FD_R	30.42 ± 1.72 ^a^	12.37 ± 1.35 ^ab^	0.59 ± 0.05 ^de^	547.89 ± 18.72 ^a^	604.85 ± 18.65 ^b^	481.24 ± 7.39 ^a^
I_FD_B	27.74 ± 1.14 ^ab^	11.48 ± 0.46 ^bc^	0.65 ± 0.03 ^d^	483.11 ± 11.44 ^b^	556.65 ± 19.30 ^c^	386.29 ± 11.37 ^b^
L_OD_R	27.03 ± 3.77 ^bc^	11.56 ± 0.20 ^b^	0.48 ± 0.06 ^f^	503.20 ± 32.28 ^b^	650.74 ± 11.63 ^a^	196.09 ± 9.34 ^d^
L_OD_B	24.67 ± 2.06 ^cd^	9.66 ± 0.42 ^d^	0.38 ± 0.06 ^g^	497.83 ± 13.65 ^b^	589.45 ± 10.19 ^b^	195.90 ± 8.36 ^d^
L_FD_R	24.08 ± 1.75 ^cd^	12.16 ± 0.43 ^ab^	0.73 ± 0.05 ^c^	474.73 ± 35.79 ^b^	528.78 ± 21.51 ^de^	241.50 ± 6.48 ^c^
L_FD_B	26.52 ± 1.34 ^bc^	10.63 ± 0.36 ^c^	0.55 ± 0.04 ^ef^	436.60 ± 22.68 ^c^	552.03 ± 14.59 ^cd^	203.49 ± 12.17 ^d^

Values are expressed as mean ± standard deviation (*n* = 9). Different lowercase letters (a–g) within each column indicate significant differences among drying and thermal treatments (one-way ANOVA, Tukey’s post hoc test, *p* < 0.05). TPC, total phenolic content; TFC, total flavonoid content; CT, condensed tannins; DPPH, 2,2-diphenyl-1-picrylhydrazyl radical scavenging activity; FRAP, ferric reducing antioxidant power; ABTS, 2,2′-azino-bis(3-ethylbenzothiazoline-6-sulfonic acid); GAE, gallic acid equivalents; CE, catechin equivalents; TE, Trolox equivalents; DE, dried extract. Samples were coded as follows: I_OD_R = oven-dried inflorescence, raw; I_OD_B = oven-dried inflorescence, boiled; I_FD_R = freeze-dried inflorescence, raw; I_FD_B = freeze-dried inflorescence, boiled; L_OD_R = oven-dried leaf, raw; L_OD_B = oven-dried leaf, boiled; L_FD_R = freeze-dried leaf, raw; and L_FD_B = freeze-dried leaf, boiled.

**Table 3:** Permutational multivariate analysis of variance (PERMANOVA) results showing the effects of tissue type, drying method, thermal treatment, and their interactions on phenolic-related variables and antioxidant capacity (TPC, TFC, CT, DPPH, FRAP, and ABTS) in *C. berlandieri* subsp. *nuttalliae*.

Source	Degrees of Freedom (df)	Sum of Squares	R^2^	Pseudo-F Value	*p*-Value
Tissue	1	66.14	0.15525	76.650	<0.001
Drying method	1	69.49	0.16311	80.532	<0.001
Thermal treatment	1	75.01	0.17608	86.934	<0.001
Tissue × Drying method	1	109.43	0.25687	126.823	<0.001
Tissue × Thermal treatment	1	16.78	0.03939	19.448	<0.001
Drying method × Thermal treatment	1	19.15	0.04495	22.194	<0.001
Tissue × Drying method × Thermal treatment	1	14.79	0.03472	17.144	<0.001
Residual	64	55.22	0.12963	NA	NA
Total	71	426.00	1.00000	NA	NA

PERMANOVA was conducted using Euclidean distance on centered and unit-variance-scaled data (z-scores). The analysis included phenolic-related colorimetric variables and antioxidant capacity parameters: total phenolic content (TPC), total flavonoid content (TFC), condensed tannins (CT), and antioxidant capacity measured by DPPH, FRAP, and ABTS assays. Statistical significance was determined using 999 permutations. Reported statistics include degrees of freedom (Df), sums of squares (Sum Sq), proportion of explained variance (R^2^), pseudo-F values, and permutation-based *p*-values.

**Table 4:** Phenolic compounds from raw and boiled oven-dried and lyophilized *C. berlandieri* ssp. *nuttalliae* leaves and inflorescences, quantified by UPLC-DAD-ESI-QToF/MS (µg of each phenolic compound/g of dried extract).

Compounds	I_FD_B	I_FD_R	I_OD_B	I_OD_R	L_FD_B	L_FD_R	L_OD_B	L_OD_R
***Flavanones***								
Naringin *	9392.0 ± 2243.6	7516.1 ± 149.4	9496.3 ± 2196.7	8152.3 ± 1885.9	5289.8 ± 1245.4	4264.3 ± 980.8	6783.3 ± 1539.3	7692.6 ± 1789.3
***Flavonols***								
(Iso)-rhamnetin glucuronide	6459.0 ± 1168.3	7099.3 ± 1356.7	N. d.	5049.7 ± 888.3	7230.0 ± 1320.2	6428.0 ± 1136.5	N. d.	6037.4 ± 1069.5
(Iso)-rhamnetin hexoside	2016.2 ± 341.2	2460.7 ± 432.4	N. d.	1836.4 ± 305.6	1989.2 ± 335.3	1721.2 ± 271.3	1149.8 ± 0.0	1531.0 ± 243.1
(Iso)-rhamnetin deoxyhexoside-hexoside	3348.5 ± 757.1	3912.8 ± 911.6	3138.8 ± 688.3	706.5 ± 153.8	1518.2 ± 304.8	203.6 ± 39.7	820.1 ± 154.9	1042.7 ± 202.4
Kaempferol aldopentoside-hexoside	215.9 ± 54.7	281.6 ± 66.0	177.3 ± 47.2	183.2 ± 43.2	551.9 ± 106.3	465.8 ± 88.6	333.7 ± 65.7	432.8 ± 92.7
Kaempferol deoxyhexoside-hexoside	213.5 ± 0.0	316.2 ± 47.0	N. d.	N. d.	885.4 ± 173.9	725.4 ± 114.8	492.3 ± 0.0	603.7 ± 23.8
Kaempferol deoxyhexoside-dihexoside	827.9 ± 216.6	969.3 ± 262.7	788.6 ± 196.0	641.4 ± 156.5	113.4 ± 30.5	92.2 ± 22.6	67.4 ± 18.4	81.2 ± 21.9
Kaempferol di-deoxyhexoside-hexoside	269.5 ± 68.6	326.8 ± 86.9	220.8 ± 58.1	206.1 ± 51.1	296.1 ± 65.7	250.6 ± 56.5	158.4 ± 37.6	205.5 ± 53.6
Kaempferol deoxyhexoside-hexoside	235.0 ± 36.5	249.8 ± 37.6	239.3 ± 36.5	158.4 ± 28.0	188.3 ± 36.7	146.7 ± 27.0	68.4 ± 15.2	77.6 ± 13.8
Kaempferol *	N. d.	N. d.	N. d.	N. d.	40.9 ± 4.0	27.2 ± 2.3	17.2 ± 1.6	N. d.
Myricetin deoxyhexoside	1086.7 ± 160.5	1547.2 ± 254.0	N. d.	1162.1 ± 165.0	1292.1 ± 196.9	1144.5 ± 165.9	807.0 ± 122.8	1042.8 ± 155.3
Quercetin aldopentoside- deoxyhexoside-hexoside	4296.7 ± 1095.7	4840.1 ± 1285.4	3758.4 ± 1025.6	3516.5 ± 825.1	409.0 ± 97.2	417.6 ± 92.8	338.7 ± 76.7	361.1 ± 89.9
Quercetin dihexoside	69.9 ± 17.2	133.4 ± 2.8	67.6 ± 16.4	76.9 ± 17.1	337.8 ± 64.6	377.3 ± 73.2	198.6 ± 37.4	350.6 ± 71.3
Quercetin glucuronide	15,726.1 ± 2798.3	17,336.5 ± 3154.0	15,112.9 ± 2697.3	13,483.5 ± 2353.9	8651.9 ± 1505.7	7824.7 ± 1359.0	6855.7 ± 1153.6	7959.1 ± 1387.6
Quercetin deoxyhexoside-dihexoside	135.6 ± 32.3	207.7 ± 40.7	130.8 ± 32.9	100.0 ± 21.3	215.6 ± 47.9	223.0 ± 49.9	N. d.	217.8 ± 55.5
Quercetin di-deoxyhexoside-hexoside	3468.4 ± 938.8	3816.4 ± 1050.8	3069.8 ± 844.8	3044.6 ± 820.9	2027.0 ± 526.1	1986.1 ± 525.9	1601.5 ± 427.9	1898.8 ± 492.7
Quercetin rutinoside *	1571.3 ± 314.4	1923.5 ± 418.9	1331.9 ± 280.2	1344.9 ± 275.4	1773.5 ± 349.6	1674.5 ± 308.0	1329.0 ± 249.4	1615.8 ± 322.5
Quercetin *	51.9 ± 4.6	59.5 ± 7.2	51.8 ± 5.2	N. d.	110.6 ± 9.7	50.1 ± 5.6	65.2 ± 6.1	27.6 ± 3.3
***Hydroxybenzoic acids***								
3,4-Dihydroxybenzoic acid *	164.8 ± 7.5	162.1 ± 7.6	182.4 ± 8.0	151.2 ± 6.6	111.3 ± 4.7	115.3 ± 4.9	110.4 ± 4.9	131.2 ± 6.0
Dihydroxybenzoic acid	N. d.	N. d.	N. d.	N. d.	12.3 ± 0.7	13.0 ± 0.8	25.9 ± 1.2	26.8 ± 1.3
Dihydroxybenzoic acid hexoside isomer I	100.2 ± 9.5	100.3 ± 9.0	121.9 ± 10.6	82.7 ± 7.2	123.5 ± 7.6	144.0 ± 9.0	196.0 ± 12.9	251.3 ± 20.8
Dihydroxybenzoic acid hexoside isomer II	1428.3 ± 151.5	1220.5 ± 134.8	1213.1 ± 133.3	797.5 ± 75.6	668.6 ± 61.7	623.9 ± 58.4	690.9 ± 71.5	752.0 ± 71.5
Caffeic acid ethyl ester	N. d.	N. d.	N. d.	N. d.	38.9 ± 2.5	51.2 ± 2.7	32.6 ± 2.1	49.0 ± 3.1
***Organic acids***								
Caffeic acid hexoside	124.8 ± 25.1	126.3 ± 24.8	111.8 ± 18.7	96.2 ± 18.4	18.1 ± 3.7	25.1 ± 9.8	22.2 ± 9.1	28.0 ± 13.2
Citric acid isomer	122.0 ± 4.9	119.2 ± 5.0	134.0 ± 5.2	63.1 ± 2.4	48.4 ± 1.7	53.4 ± 1.7	47.1 ± 1.8	53.6 ± 2.1
Citric acid *	193.1 ± 8.3	195.4 ± 8.2	208.3 ± 9.1	227.6 ± 9.2	104.4 ± 4.3	128.6 ± 4.5	235.5 ± 8.7	239.1 ± 6.7

Values are expressed as mean ± standard deviation (*n* = 3). Compounds marked with (*) were confirmed using reference standards (MSI level 1), whereas the remaining compounds were tentatively identified based on spectral information and database comparison (MSI level 2). Analyses were performed in negative electrospray ionization mode ([M–H]^−^). Samples were coded as follows: I_OD_R = oven-dried inflorescence, raw; I_OD_B = oven-dried inflorescence, boiled; I_FD_R = freeze-dried inflorescence, raw; I_FD_B = freeze-dried inflorescence, boiled; L_OD_R = oven-dried leaf, raw; L_OD_B = oven-dried leaf, boiled; L_FD_R = freeze-dried leaf, raw; and L_FD_B = freeze-dried leaf, boiled.

## Data Availability

Data is contained within the article or Supplementary Material.

## Supplementary Materials

The following supporting information can be downloaded at: https://www.mdpi.com/article/10.3390/plants15091366/s1, Table S1. Phenolic compounds identified in inflorescences and leaves of *C. berlandieri* subsp. *nuttalliae* by UPLC–DAD–ESI–QToF/MS. Table S2. Calibration curve equations for the quantification of phenolic compounds by UPLC–DAD–ESI–QToF/MS, including slope, coefficient of determination (R^2^), limit of detection (LOD), and limit of quantification (LOQ) for each reference standard. Table S3. Explained and cumulative variance of principal component analysis (PCA) based on colorimetric phenolic variables and antioxidant capacity in inflorescence and leaf tissues of *C. berlandieri* subsp. *nuttalliae*. Table S4. Principal component loadings for colorimetric phenolic variables and antioxidant capacity (TPC, TFC, CT, DPPH, FRAP, and ABTS) in *C. berlandieri* subsp. *nuttalliae*. Table S5. Sample scores for the first three principal components (PC1–PC3) derived from PCA of colorimetric phenolic variables in *C. berlandieri* subsp. *nuttalliae*. Table S6. PERMDISP test for homogeneity of multivariate dispersion among experimental groups based on standardized colorimetric phenolic variables and antioxidant capacity (Euclidean distance). Table S7. Topological parameters of phenolic metabolic co-occurrence networks in inflorescence and leaf tissues of *C. berlandieri* subsp. *nuttalliae*. Table S8. Explained and cumulative variance of principal component analysis (PCA) based on phenolic metabolite profiles identified by UPLC–DAD–ESI–QToF/MS in *C. berlandieri* subsp. *nuttalliae*. Table S9. Principal component loadings for phenolic metabolites identified by UPLC–DAD–ESI–QToF/MS in *C. berlandieri* subsp. *nuttalliae*. Table S10. Sample scores (PC1–PC3) derived from PCA of phenolic metabolite profiles identified by UPLC–DAD–ESI–QToF/MS in *C. berlandieri* subsp. *nuttalliae*. Figure S1. Representative chromatographic profiles of phenolic compounds in *C. berlandieri* subsp. *nuttalliae* under different processing conditions. Figure S2. High- and low-energy ESI–QToF MS spectra of naringin identified in *C. berlandieri* subsp. *nuttalliae* based on accurate mass, retention time, and MS/MS fragmentation patterns. Figure S3. High- and low-energy ESI–QToF MS spectra of a quercetin glycoside (di-deoxyhexoside–hexoside) tentatively identified in *C. berlandieri* subsp. *nuttalliae* based on accurate mass and MS/MS fragmentation patterns. Figure S4. High- and low-energy ESI–QToF MS spectra of a quercetin glycoside (aldopentoside–deoxyhexoside–hexoside) tentatively identified in *C. berlandieri* subsp. *nuttalliae* based on accurate mass and MS/MS fragmentation patterns. Figure S5. High- and low-energy ESI–QToF MS spectra of an (iso)-rhamnetin glycoside (deoxyhexoside–hexoside) tentatively identified in *C. berlandieri* subsp. *nuttalliae* based on accurate mass and MS/MS fragmentation patterns. Figure S6. High- and low-energy ESI–QToF MS spectra of an (iso)-rhamnetin glucuronide tentatively identified in *C. berlandieri* subsp. *nuttalliae* based on accurate mass and MS/MS fragmentation patterns.

## Abbreviations

The following abbreviations are used in this manuscript:

ABTS	2,2′-Azino-bis(3-ethylbenzothiazoline)-6-sulfonic acid
ANOVA	Analysis of variance
AOAC	Association of Official Analytical Chemists
CE	Catechin equivalents
CT	Condensed tannins
DAD	Diode array detector
DE	Dried extract
DPPH	1,1-Diphenyl-2-picrylhydrazyl
ESI	Electrospray ionization
FRAP	Ferric reducing antioxidant power
GAE	Gallic acid equivalents
MS	Mass spectrometry
PCA	Principal component analysis
PERMANOVA	Permutational multivariate analysis of variance
PERMDISP	Permutational analysis of multivariate dispersions
PLS-DA	Partial least squares discriminant analysis
QToF	Quadrupole time-of-flight
TFC	Total flavonoid content
TPC	Total phenolic content
UPLC	Ultra-performance liquid chromatography
VIP	Variable importance in projection
