# Supplementary material for: Effect of Drying and Cooking on the Chemical Composition, Phenolic Profile, and Antioxidant Capacity of Chenopodium berlandieri subsp. nuttalliae: A Metabolomic Approach
Source: Plants (Basel). 2026 Apr 29;15(9):1366. doi: 10.3390/plants15091366 (PMC13164582; doi:10.3390/plants15091366)
Supplement: Supplementary file 1 [file plants-15-01366-s001.zip › plants-4224767-supplementary.pdf]

Supplementary Material

Effect of Drying and Cooking on the Chemical Composition, Phenolic Profile, and Antioxidant Capacity of *Chenopodium berlandieri* subsp. *nuttalliae*: A Metabolomic Approach

Supplementary Table S1. Phenolic compounds identified in inflorescences and leaves of *C. berlandieri* subsp. *nuttalliae* by UPLC–DAD–ESI–QToF/MS.

Class	Identified compound	RT (min)	Molecular formula	Expected mass (Da)	Observed mass (Da)	Observed m/z	Mass error (ppm)	Adducts	MS/MS fragments
Flavanones	Naringin*	7.28	$C_{27}H_{32}O_{14}$	580.1792	580.1816	579.1697	4.0835	$[M{-}H]^-$	459.11453, 151.00332
Flavonols	Quercetin dihexoside	2.52	$C_{27}H_{30}O_{17}$	626.1483	626.1488	625.1415	0.8005	$[M{-}H]^-$	301.03504, 151.0410
	Quercetin deoxyhexoside-dihexoside	2.56	$C_{33}H_{40}O_{21}$	772.2062	772.2068	771.1995	0.7386	$[M{-}H]^-$	301.03505, 151.00418
	Quercetin di-deoxyhexoside-hexoside	3.63	$C_{33}H_{40}O_{20}$	756.2113	756.2123	755.2050	1.3380	$[M{-}H]^-$	300.02756, 151.00407, 107.01442
	Quercetin aldopentoside-deoxyheoside-hexoside	3.89	$C_{32}H_{38}O_{20}$	742.1956	742.1965	741.1893	1.1947	$[M{-}H]^-$	300.02780, 151.03659, 107.01959
	Kaempferol deoxyheoside-dihexoside	3.93	$C_{33}H_{40}O_{20}$	756.2113	756.2140	755.2067	3.5185	$[M{-}H]^-$	284.0337, 151.07266
	Kaempferol di-deoxyheoside-hexoside	4.30	$C_{33}H_{40}O_{19}$	740.2164	740.2188	739.2116	3.3186	$[M{-}H]^-$	284.02456, 151.04082

Quercetin rutinoside*	4.52	$C_{27}H_{30}O_{16}$	610.1534	610.1539	609.1467	0.9078	$[M-H]^-$	400.02788, 151.09339
Quercetin glucuronide	4.67	$C_{21}H_{18}O_{13}$	478.0747	478.0756	477.0683	1.7812	$[M-H]^-$	301.03602, 151.00432, 107.01440
Myricetin deoxyhexoside	4.81	$C_{21}H_{20}O_{12}$	464.0955	464.0967	463.0895	2.7042	$[M-H]^-$	317.08697, 300.02825, 151.11360
Kaempferol deoxyhexoside-hexoside	4.86	$C_{27}H_{30}O_{15}$	594.1585	594.1602	593.1529	2.8804	$[M-H]^-$	284.03223, 151.11358
Kaempferol aldopentoside-hexoside	5.23	$C_{26}H_{28}O_{15}$	580.1428	580.1448	579.1375	3.4518	$[M-H]^-$	284.03252, 151.02477
(Iso)-rhamnetin deoxyhexoside-hexoside	6.21	$C_{28}H_{32}O_{16}$	624.1690	624.1668	623.1596	-3.5405	$[M-H]^-$	315.05143, 300.02795
(Iso)-rhamnetin hexoside	6.56	$C_{22}H_{22}O_{12}$	478.1111	478.1112	477.1039	0.0818	$[M-H]^-$	314.04368, 151.01811
(Iso)-rhamnetin glucuronide	6.73	$C_{22}H_{20}O_{13}$	492.0904	492.0904	491.0832	0.1117	$[M-H]^-$	315.05125, 300.02784, 151.00416
Kaempferol deoxyhexoside-hexoside	7.34	$C_{27}H_{30}O_{15}$	594.1585	594.1584	593.1511	-0.1665	$[M-H]^-$	284.05578, 151.01168
Quercetin*	10.16	$C_{15}H_{10}O_7$	302.0427	302.0428	301.0355	0.5375	$[M-H]^-$	178.9981, 151.00395, 107.01397
Kaempferol*	11.24	$C_{15}H_{10}O_6$	286.0477	286.0486	285.0413	2.9281	$[M-H]^-$	151.0039, 107.01407

Hydroxybenzoic acids	Dihydroxybenzoic acid hexoside isomer I	1.38	$C_{13}H_{16}O_9$	316.0794	316.0791	315.0718	-1.1147	$[M–H]^-$	153.01971, 134.03753, 108.02161
	Dihydroxybenzoic acid hexoside isomer II	1.93	$C_{13}H_{16}O_9$	316.0794	316.0793	315.0720	-0.3401	$[M–H]^-$	153.01920, 135.00901, 109.02936
	Dihydroxybenzoic acid	2.26	$C_7H_6O_4$	154.0266	154.0267	153.0195	0.8153	$[M–H]^-$	135.02437, 109.02940
	3,4-Dihydroxybenzoic acid*	3.39	$C_7H_6O_4$	154.0266	154.0270	153.0197	2.7249	$[M–H]^-$	135.00909, 109.02989
	Caffeic acid hexoside	2.48	$C_{15}H_{18}O_9$	342.0951	342.0946	341.0873	-1.3860	$[M–H]^-$	179.03527, 135.04538
	Caffeic acid ethyl ester	2.73	$C_{11}H_{12}O_4$	208.0736	208.0736	207.0663	0.2215	$[M–H]^-$	179.05616, 135.02923
Organic acids	Citric acid*	0.65	$C_6H_8O_7$	192.0270	192.0270	191.0197	-0.0784	$[M–H]^-$	111.00929
	Citric acid isomer	2.08	$C_6H_8O_7$	192.0270	192.0270	191.0197	-0.1672	$[M–H]^-$	111.0077

RT: retention time; m/z: mass-to-charge ratio; ppm: mass error expressed as parts per million. Phenolic compounds were identified using UPLC–DAD–ESI–QTOF/MS based on accurate mass measurements, molecular formula prediction, MS/MS fragmentation patterns, and comparison with previously reported phenolic compounds. Mass errors were within ±5 ppm. Compounds marked with (*) were confirmed using reference standards (MSI level 1), whereas the remaining compounds were tentatively identified based on spectral information and database comparison (MSI level 2). Analyses were performed in negative electrospray ionization mode ($[M–H]^-$). For compounds without authentic standards, annotations refer to putative structures based on mass spectral data and neutral losses, and do not imply confirmed sugar identity or linkage.

Supplementary Table S2. Calibration curve equations for the quantification of phenolic compounds by UPLC–DAD–ESI–QToF/MS, including slope, coefficient of determination (R^2), limit of detection (LOD), and limit of quantification (LOQ) for each reference standard.

**Standard**	**Slope**	**R2 coefficient**	**LOD (ng/mL)**	**LOQ (ng/mL)**	**Quantified compounds**
Naringin	4641741.08	0.9981	0.01	0.04	Naringin
Quercetin	4912823.68	0.9977	0.02	0.05	Quercetin and its derivatives, (iso)-rhamnetin derivatives, and myricetin derivatives
Quercetin rutinoside	6455696.62	0.9981	0.02	0.06	Quercetin rutinoside
Kaempferol	14467384.40	0.9992	0.01	0.02	Kaempferol and its derivatives
3,4-Dihydroxybenzoic acid	484291.48	0.9987	0.01	0.04	Dihydroxybenzoic acids
Caffeic acid	3451173.30	0.9992	0.01	0.02	Caffeic acid derivatives
Citric acid	4183.14	0.9834	0.01	0.03	Citric acid and its isomer

Calibration curves were constructed from six different concentrations for each standard and were analyzed by triplicate. LOD: limit of detection; LOQ: limit of quantification.

Supplementary Table S3. Explained and cumulative variance of principal component analysis (PCA) based on colorimetric phenolic variables and antioxidant capacity in inflorescence and leaf tissues of *C. berlandieri* subsp. *nuttalliae*

Inflorescence		
**Principal component (PCA)**	**Percentage (%)**	**Cumulative Percentage (%)**
PC1	60.135	60.135
PC2	27.854	87.990
PC3	7.195	95.185
PC4	3.413	98.599
PC5	1.087	99.686
PC6	0.314	100.00
**Leaf**		
**Principal component (PCA)**	**Percentage (%)**	**Cumulative Percentage (%)**
PC1	48.862	48.862
PC2	21.2960	70.158
PC3	16.873	87.031
PC4	7.855	94.886
PC5	2.902	97.788
PC6	2.212	100.00

PCA was performed on centered and unit-variance-scaled variables using mean values of three replicates (n = 9) per treatment.

Supplementary Table S4. Principal component loadings for colorimetric phenolic variables and antioxidant capacity (TPC, TFC, CT, DPPH, FRAP, and ABTS) in *C. berlandieri* subsp. *nuttalliae*.

			**Inflorescence**			
**Variable**	**PC1**	**PC2**	**PC3**	**PC4**	**PC5**	**PC6**
TPC	-0.4990	-0.1186	-0.1088	0.4543	-0.6704	0.2631
TFC	-0.4110	0.3431	-0.6368	0.0881	0.4979	0.2284
CT	0.2806	0.6174	-0.3738	-0.0643	-0.4667	-0.4223
DPPH	-0.1543	0.6530	0.6399	0.3363	0.1502	0.0684
FRAP	-0.5077	-0.1771	0.0617	0.0975	0.1423	-0.8230
ABTS	-0.4709	0.1712	0.1720	-0.8119	-0.2049	0.1350
			**Leaf**			
**Variable**	**PC1**	**PC2**	**PC3**	**PC4**	**PC5**	**PC6**
TPC	0.1770	0.4002	-0.7729	0.4569	0.0315	-0.0368
TFC	-0.39787	0.5494	-0.1007	-0.4538	0.0290	0.5683
CT	-0.5376	0.1823	0.0045	0.0653	-0.6762	-0.4650
DPPH	0.2364	0.5106	0.6061	0.5112	-0.1305	0.1942
FRAP	-0.5294	0.1473	0.1414	0.2390	0.7139	-0.3336
ABTS	0.4305	0.4716	0.0712	-0.5125	0.1190	-0.5572

PCA was performed on centered and unit-variance-scaled variables using mean values of three replicates (n = 9) per treatment.

Supplementary Table S5. Sample scores for the first three principal components (PC1–PC3) derived from PCA of colorimetric phenolic variables in *C. berlandieri* subsp. *nuttalliae*.

**Sample**	**PC1**	**PC2**	**PC3**
I_OD_R	0.9473	-0.3721	0.9898
I_OD_B	3.2046	0.9273	0.3028
I_FD_R	-1.6422	-1.2078	1.4260
I_FD_B	-0.1096	-0.9480	-0.0085
L_OD_R	-1.4490	1.1504	0.0421
L_OD_B	-0.7546	1.8468	0.0686
L_FD_R	0.7371	-1.7658	-1.2495
L_FD_B	-0.9336	0.3691	-1.5711

PCA was performed on centered and unit-variance-scaled variables using mean values of three replicates (n = 3). Samples were coded as follows: I_OD_R = oven-dried inflorescence, raw; I_OD_B = oven-dried inflorescence, boiled; I_FD_R = freeze-dried inflorescence, raw; I_FD_B = freeze-dried

inflorescence, boiled; L_OD_R = oven-dried leaf, raw; L_OD_B = oven-dried leaf, boiled; L_FD_R = freeze-dried leaf, raw; and L_FD_B = freeze-dried leaf, boiled.

Supplementary Table S6. PERMDISP test for homogeneity of multivariate dispersion among experimental groups based on standardized colorimetric phenolic variables and antioxidant capacity (Euclidean distance).

Source	Df	Sum Sq	Mean Sq	F value	Pr(>F)
Groups	7	1.5220	0.21742	1.0495	0.4063
Residuals	64	13.2593	0.20718	NA	NA

PERMDISP evaluates homogeneity of multivariate dispersions (i.e., within-group variability) among groups using betadisper; Euclidean distances were computed on centered and unit-variance-scaled variables, and significance was assessed by permutation (999 permutations). A non-significant p-value indicates no evidence of unequal dispersion among groups.

Supplementary Table S7. Topological parameters of phenolic metabolic co-occurrence networks in inflorescence and leaf tissues of *C. berlandieri* subsp. *nuttalliae*.

**Inflorescence**			
**Metabolite**	**Degree**	**Betweenness**	**Module**
Naringin*	0	0	1
Quercetin dihexoside	10	0.021	2
Quercetin deoxyheoside-dihexoside	14	0.038	2
Quercetin di-deoxyheoside-hexoside	14	0.012	3
Quercetin aldopentoside deoxyhexoside-hexoside	14	0.012	3
Kaempferol deoxyheoside-dihexoside	14	0	3
Kaempferol di-deoxyheoside-hexoside	14	0.005	3
Quercetin rutinoside*	13	0	3
Quercetin glucuronide	14	0.012	3
Myricetin deoxyhexoside	2	0	2
Kaempferol deoxyhexoside-hexoside	0	0	4
Kaempferol aldopentoside hexoside	13	0	3
(Iso)-rhamnetin deoxyhexoside-hexoside	14	0.120	3
(Iso)-rhamnetin hexoside	14	0.003	3
(Iso)-rhamnetin glucuronide	14	0.003	3
Kaempferol deoxyhexoside-hexoside	14	0.161	5
Quercetin*	12	0	3
Kaempferol*	0	0	6
Dihydroxybenzoic acid hexoside isomer I	5	0	5

Dihydroxybenzoic acid hexoside isomer II	2	0.063	7
Dihydroxybenzoic acid	0	0	8
3,4-Dihydroxybenzoic acid *	3	0	5
Caffeic acid hexoside	14	0.012	3
Caffeic acid ethyl ester	0	0	9
Citric acid*	1	0	7
Citric acid isomer	3	0	5
**Leaf**			
**Metabolite**	**Degree**	**Betweenness**	**Module**
Naringin*	4	0.033	1
Quercetin dihexoside	12	0.190	2
Quercetin deoxyhexoside-dihexoside	2	0	2
Quercetin di-deoxyhexoside-hexoside	11	0.013	3
Quercetin aldopentoside-deoxyheoside-hexoside	12	0.040	3
Kaempferol deoxyhexoside-dihexoside	12	0.022	3
Kaempferol di-deoxyhexoside-hexoside	13	0.048	3
Quercetin rutinoside*	12	0.022	3
Quercetin glucuronide	9	0.063	3
Myricetin deoxyhexoside	13	0.032	3
Kaempferol deoxyheoside-dihexoside	7	0.090	3
Kaempferol aldopentoside hexoside	13	0.016	3
(Iso)-rhamnetin deoxyheoside-dihexoside	0	0	4
(Iso)-rhamnetin hexoside	11	0.020	3
(Iso)-rhamnetin glucuronide	10	0	3
Kaempferol deoxyheoside-hexoside	11	0.020	3
Quercetin*	1	0	1
Kaempferol*	10	0.120	3
Dihydroxybenzoic acid hexoside isomer I	5	0.100	1
Dihydroxybenzoic acid hexoside isomer II	3	0.073	1
Dihydroxybenzoic acid	4	0	1
3,4-Dihydroxybenzoic acid *	4	0.033	2
Caffeic acid hexoside	5	0.130	2
Caffeic acid ethyl ester	3	0	2
Citric acid*	5	0.060	1
Citric acid isomer	4	0	2

Topological parameters of the phenolic metabolic co-occurrence networks constructed for inflorescence and leaf tissues *of C. berlandieri* subsp. *nuttalliae*. Degree represents the number of significant correlations for each metabolite within the metabolic network ($|\varrho| \geq 0.70$; $p \leq 0.05$). The networks were constructed using 26 metabolites detected in each tissue. Betweenness centrality indicates the extent to which a metabolite acts as an intermediary node connecting different parts of the network. Module corresponds to communities detected using the Louvain algorithm, grouping metabolites with similar co-accumulation patterns.

Supplementary Table S8. Explained and cumulative variance of principal component analysis (PCA) based on phenolic metabolite profiles identified by UPLC–DAD–ESI–QToF/MS in *C. berlandieri* subsp. *nuttalliae*.

Component	Individual variance (%)	Cumulative variance (%)
PC1	56.0	56.0
PC2	25.0	81.0
PC3	8.3	89.3
PC4	5.8	95.1
PC5	3.6	98.7
PC6	0.9	99.6
PC7	0.4	100

PCA was performed on centered and unit-variance-scaled variables using mean values of three replicates (n = 3).

Supplementary Table S9. Principal component loadings for phenolic metabolites identified by UPLC–DAD–ESI–QToF/MS in *C. berlandieri* subsp. *nuttalliae*.

Metabolite	Loading PC1	Loading PC2
Caffeic_acid_ethyl_ester	0.25183950	0.01548278
Quercetin_dihexoside	0.23943084	0.10799295
Kaempferol_aldopent_hex	0.23127537	0.16630243
Kaempferol_hex_rham	0.22917047	0.16756197
DHB_acid	0.22080611	-0.13154427
Kaempferol	0.19448255	0.14238833
DHB_acid_hexoside_I	0.16968814	-0.16315858
Quercetin	0.08323567	0.18421803
Myricetin_rhamnoside	0.05775016	0.27700479
Quercetin_hex_rham_hex	0.04060954	0.29584878

PCA was performed on centered and unit-variance-scaled variables using mean values of three replicates (n = 3).

Supplementary Table S10. Sample scores (PC1–PC3) derived from PCA of phenolic metabolite profiles identified by UPLC–DAD–ESI–QToF/MS in *C. berlandieri* subsp. *nuttalliae*.

Sample	Score_PC1	Score_PC2	Score_PC3
I_FD_R	-3.707233063	3.24690169	0.794758102
I_FD_B	-4.032818986	0.931900131	0.181535692
I_OD_R	-2.10487459	-1.472736699	1.809028705
I_OD_B	-4.19538179	-2.417412579	-2.366481741
L_FD_R	3.939705063	1.407421395	-0.19808432
L_FD_B	3.835042647	3.123291204	-1.548958019
L_OD_R	3.18766567	-1.238664646	1.736229956
L_OD_B	3.077895048	-3.580700495	-0.408028375

PCA was performed on centered and unit-variance-scaled variables using mean values of three replicates (n = 3).

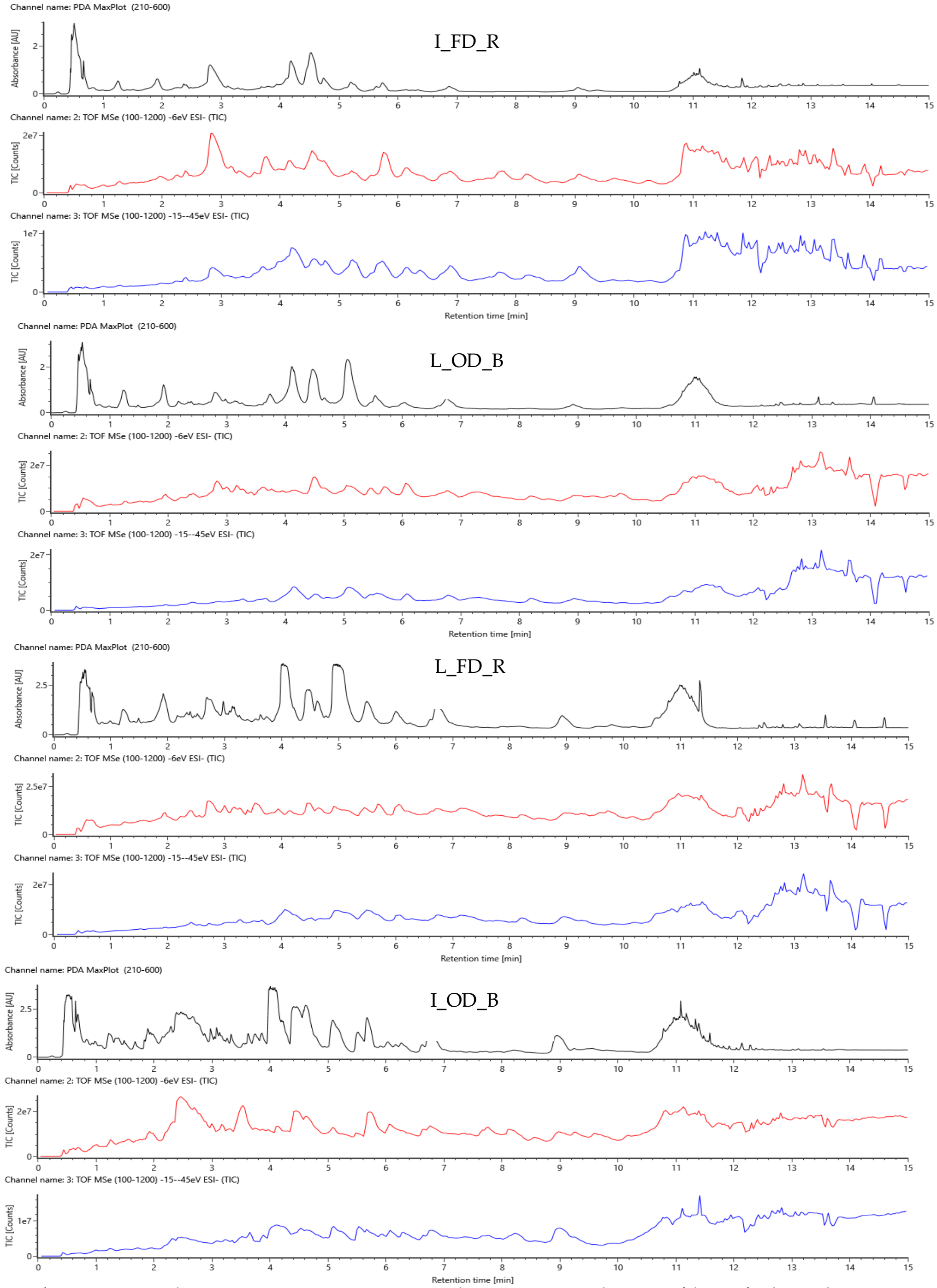

Suplementary Figure S1. Representative chromatographic profiles of phenolic compounds in *Chenopodium berlandieri* subsp. *nuttalliae* under different processing conditions.

PDA (210–600 nm) and ESI–QToF MSe total ion chromatograms (low and high collision energy) of selected samples, including I_FD_R (raw freeze-dried inflorescences), I_OD_B (boiled oven-dried inflorescences), L_FD_R (raw freeze-dried leaves), and L_OD_B (boiled oven-dried leaves). These representative chromatograms illustrate the influence of tissue type (inflorescences vs. leaves),

drying method (freeze-drying vs. oven-drying), and thermal treatment (raw vs. boiled) on the phenolic profile of the samples.

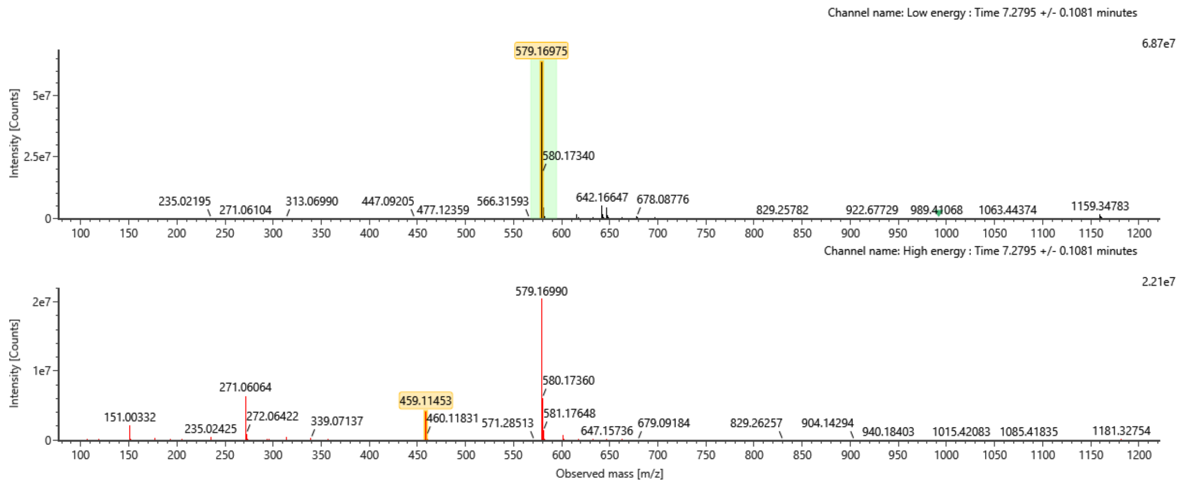

Supplementary Figure S2. High- and low-energy ESI–QToF MS spectra of naringin identified in *Chenopodium berlandieri* subsp. *nuttalliae* based on accurate mass, retention time, and MS/MS fragmentation patterns.

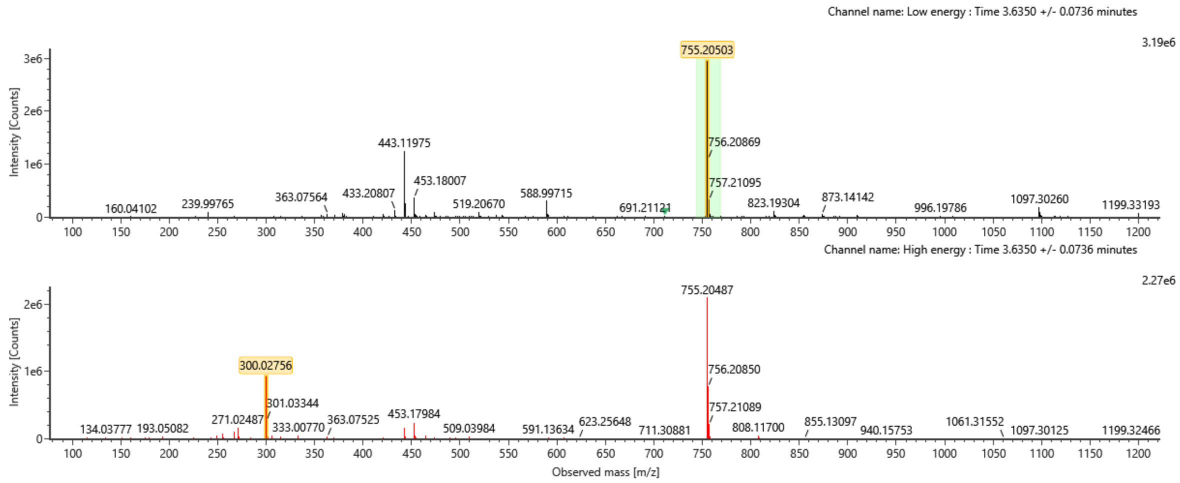

Supplementary Figure S3. High- and low-energy ESI–QToF MS spectra of a quercetin glycoside (di-deoxyhexoside–hexoside) tentatively identified in *Chenopodium berlandieri* subsp. *nuttalliae* based on accurate mass and MS/MS fragmentation patterns.

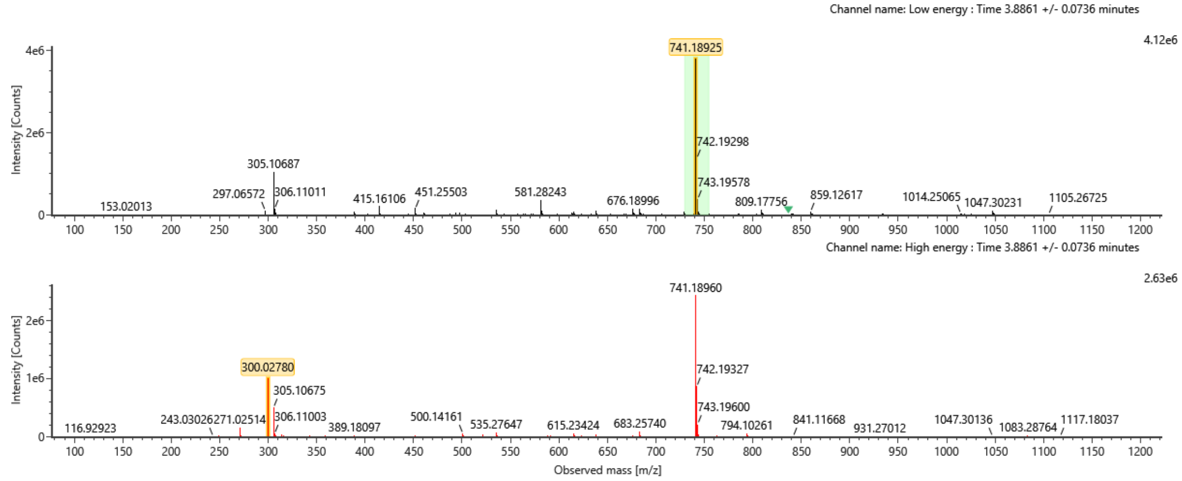

Supplementary Figure S4. High- and low-energy ESI–QToF MS spectra of a quercetin glycoside (aldopentoside–deoxyhexoside–hexoside) tentatively identified in *Chenopodium berlandieri* subsp. *nuttalliae* based on accurate mass and MS/MS fragmentation patterns.

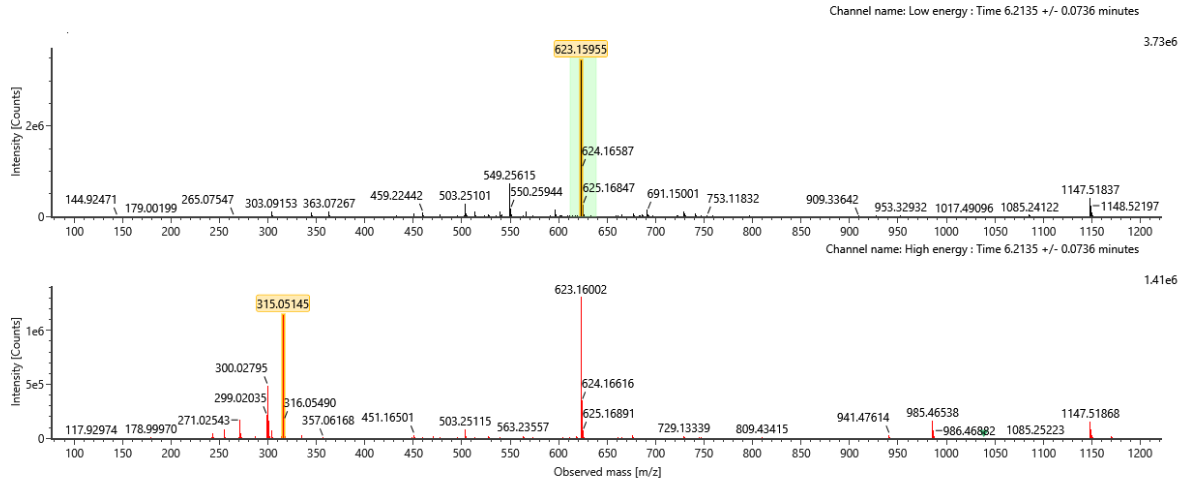

Supplementary Figure S5. High- and low-energy ESI–QToF MS spectra of an (iso)-rhamnetin glycoside (deoxyhexoside–hexoside) tentatively identified in *Chenopodium berlandieri* subsp. *nuttalliae* based on accurate mass and MS/MS fragmentation patterns.

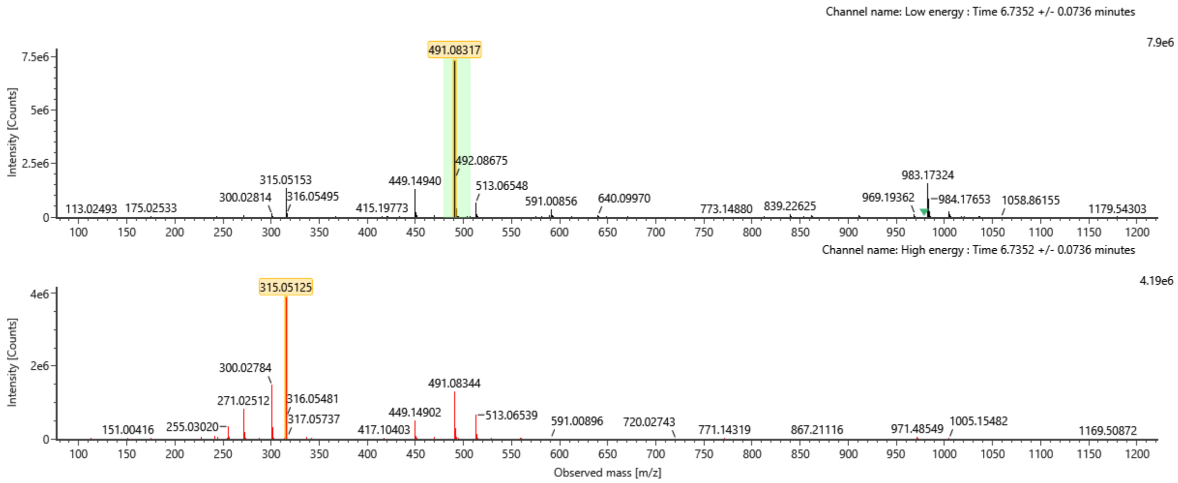

Supplementary Figure S6. High- and low-energy ESI–QToF MS spectra of an (iso)-rhamnetin glucuronide tentatively identified in *Chenopodium berlandieri* subsp. *nuttalliae* based on accurate mass and MS/MS fragmentation patterns.
